# Multiparameter functional diversity of human C2H2 zinc finger proteins

**DOI:** 10.1101/gr.209643.116

**Published:** 2016-12

**Authors:** Frank W. Schmitges, Ernest Radovani, Hamed S. Najafabadi, Marjan Barazandeh, Laura F. Campitelli, Yimeng Yin, Arttu Jolma, Guoqing Zhong, Hongbo Guo, Tharsan Kanagalingam, Wei F. Dai, Jussi Taipale, Andrew Emili, Jack F. Greenblatt, Timothy R. Hughes

**Affiliations:** 1 Donnelly Centre for Cellular and Biomolecular Research, University of Toronto, Toronto, Ontario M5S 3E1, Canada;; 2 Department of Molecular Genetics, University of Toronto, Toronto, Ontario M5S 1A8, Canada;; 3 Department of Biosciences and Nutrition, Karolinska Institutet, SE 141 83, Sweden

## Abstract

C2H2 zinc finger proteins represent the largest and most enigmatic class of human transcription factors. Their C2H2-ZF arrays are highly variable, indicating that most will have unique DNA binding motifs. However, most of the binding motifs have not been directly determined. In addition, little is known about whether or how these proteins regulate transcription. Most of the ∼700 human C2H2-ZF proteins also contain at least one KRAB, SCAN, BTB, or SET domain, suggesting that they may have common interacting partners and/or effector functions. Here, we report a multifaceted functional analysis of 131 human C2H2-ZF proteins, encompassing DNA binding sites, interacting proteins, and transcriptional response to genetic perturbation. We confirm the expected diversity in DNA binding motifs and genomic binding sites, and provide motif models for 78 previously uncharacterized C2H2-ZF proteins, most of which are unique. Surprisingly, the diversity in protein–protein interactions is nearly as high as diversity in DNA binding motifs: Most C2H2-ZF proteins interact with a unique spectrum of co-activators and co-repressors. Thus, multiparameter diversification likely underlies the evolutionary success of this large class of human proteins.

Transcription factors (TFs) bind to specific DNA sequences and regulate transcription (Latchman 2008). TFs are characterized by the presence of a DNA-binding domain (DBD), and may also contain effector domains that mediate interactions with cofactors. The repertoire of TFs varies drastically across eukaryotes; for example, the nuclear receptor family has expanded dramatically in nematodes, while in vertebrates, the C2H2 zinc finger (C2H2-ZF) family is the most numerous and diverse, comprising over 700 of the ∼1600 putative human TFs (Weirauch and Hughes 2011). Diversification of TF functions can be driven by alteration of DNA sequence specificity, protein–protein interactions (PPIs), and the expression pattern of the TF-encoding gene. All three parameters contribute to divergence within the *Caenorhabditis elegans* bHLH TF family (Grove et al. 2009), but it is largely unknown whether this is the case in other TF families, and to what extent.

C2H2-ZF proteins are characterized by tandem arrays of C2H2-ZF domains, which mediate DNA interaction. The C2H2-ZF domains each contact three or more bases, with the fingers binding sequentially, such that the motifs preferred by a C2H2-ZF array typically resemble concatenation of the base preferences for the individual C2H2-ZF domains (Wolfe et al. 2000). These sequence preferences often correspond to the identity of “specificity residues” at amino acid (AA) positions -1, 2, 3, and 6 of the DNA-contacting alpha helix. The sequence specificities of C2H2-ZF proteins are highly variable (Najafabadi et al. 2015b), with many human C2H2-ZF proteins having a unique set of DNA-contacting specificity residues. The modular fashion of DNA recognition by C2H2-ZF proteins facilitates adaptation, which occurs on relatively short evolutionary timescales (Emerson and Thomas 2009).

C2H2-ZF proteins also often harbor one or more of a small number of effector domains. Roughly half (∼350) of the human C2H2-ZF proteins contain a Krüppel-Associated Box (KRAB) domain. KRAB is a small, largely unstructured domain (Mannini et al. 2006) that is best known for recruiting TRIM28 (also called KAP1) and thereby repressing transcription by subsequent recruitment of SETDB1, a histone H3 lysine 9 (H3K9) trimethylase (Schultz et al. 2002). The involvement of TRIM28 in silencing endogenous retroelements (ERE) has led to the theory that KRAB-domain C2H2-ZF proteins evolve rapidly in order to silence EREs (Matsui et al. 2010; Rowe and Trono 2011). Consistent with this notion, many human KRAB-containing C2H2-ZF proteins bind specific ERE subtypes (Najafabadi et al. 2015b), but most remain functionally unstudied. The fact that the KRAB-C2H2-ZF genes display widely varying expression patterns suggests that they take on other host functions (Huntley et al. 2006; Corsinotti et al. 2013). Indeed, there are a few reported examples of KRAB-C2H2-ZF proteins with potential host functions; ZFP809, for example, silences retroviral DNAs in mouse ES cells, but there is only partial overlap of ZFP809 binding sites with H3K9me3 in these cells (Wolf and Goff 2009).

In addition, ∼52 human C2H2-ZF proteins contain a BTB domain, and ∼50 a SCAN domain, both of which form homo- or heterodimers with other BTB or SCAN proteins, respectively (Schumacher et al. 2000; Perez-Torrado et al. 2006). Despite their highly conserved structures, both BTB and SCAN domains are very selective in their choice of dimerization partners, allowing them to recruit a distinct set of cofactors (Collins et al. 2001). Other domains are also found in a small number of human C2H2-ZF proteins (e.g., 12 contain a SET domain), but over 200 human C2H2-ZF proteins contain no conserved domains other than the C2H2-ZF array. This latter group encompasses a number of highly conserved proteins with established functional PPIs. CTCF, for example, recruits a context-dependent set of cofactors and fulfills a variety of functions, including both gene activation and repression, chromatin insulation, genomic imprinting, and genome topology (Ladomery and Dellaire 2002). Members of the Krüppel-like factor (KLF) subfamily of C2H2-ZF proteins have a highly conserved set of three zinc fingers on their carboxyl end that recognizes the GT box motif, but the N-terminus varies among KLF members and allows distinct PPIs and distinct functions (Bieker 2001).

The fact that most C2H2-ZF proteins contain an effector domain that mediates specific PPIs suggests that the family evolves primarily by alteration of DNA binding, while effector function varies only within a small spectrum of possible interaction partners. This possibility has not been tested, however. To better understand the functions and evolutionary expansion of the C2H2-ZF family, we undertook a systematic multifaceted analysis of an unbiased set of 131 human C2H2-ZF proteins, taking advantage of the compatibility of inducible, tagged constructs with multiple assays.

## Results

### C2H2-ZF proteins often bind outside of open chromatin

We previously described analysis of 39 human C2H2-ZF proteins using ChIP-seq with inducible GFP-tagged constructs in HEK293 cells (Najafabadi et al. 2015b). This uniform experimental system removes potentially confounding variables such as cell type, affinity tag, and lack of expression. A caveat of heterologous expression is that, by definition, it represents overexpression; thus, it is possible that an expanded range of binding events may be observed, relative to physiological levels. In a previous study, however, we found that protein induction levels were comparable to endogenous protein levels (Marcon et al. 2014). This system also facilitates motif derivation; both known and novel motifs, even in repetitive sequence, can be identified on the basis of consistency with the “recognition code,” which predicts binding motifs using the specificity residues (Najafabadi et al. 2015a).

Here, we expanded this approach to 131 C2H2-ZF proteins, selected to encompass diverse aspects of the C2H2-ZF family (Supplemental Table S1; Supplemental Fig. S1). These proteins represent all major C2H2-ZF subfamilies (KRAB [55], SCAN [8], KRAB + SCAN [4], BTB [9], SET [2], and no defined auxiliary domain [53]). The 131 proteins span molecular ages from primate-specific to deep metazoan origin, and the number of C2H2-ZF domains ranges from 3 to 21 (Supplemental Table S2). We included 25 C2H2-ZF proteins with an established motif (e.g., CTCF, YY1) (not counting those we previously published [Najafabadi et al. 2015b]); the majority of the proteins (78/131) had no previous motif from any source, however. Peak numbers, proportion of peaks with motifs, motif enrichment, motif centrality, and other parameters of the ChIP-seq analysis are documented in Supplemental Table S3.

**Figure 1.**
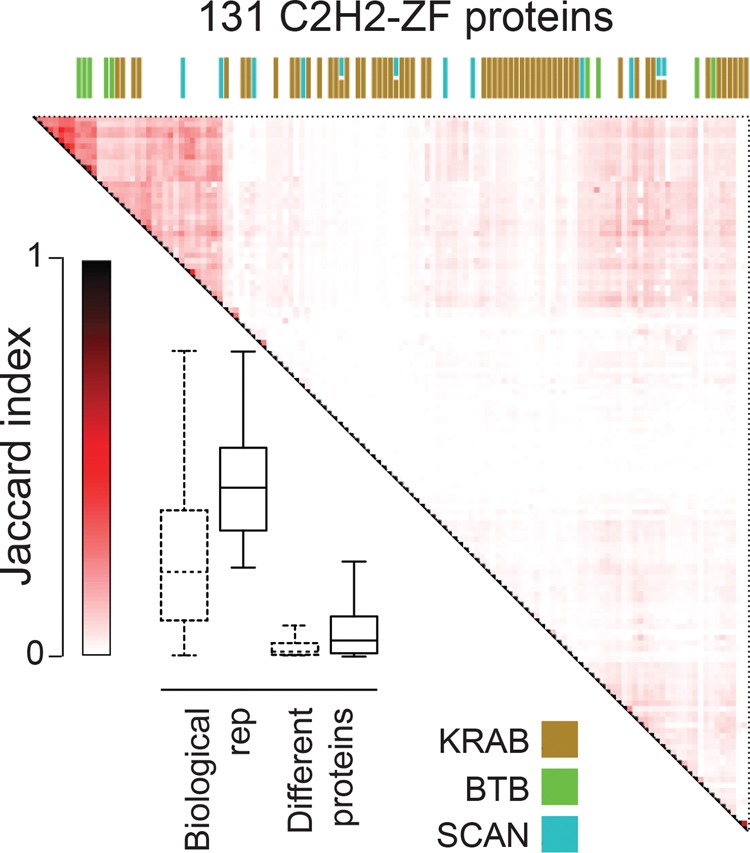
ChIP-seq analysis of 131 human C2H2-ZF proteins, shown as Jaccard similarity among different proteins, as well as between replicates. The heat map shows Jaccard similarity for binding sites of pairs of proteins, with the maximum Jaccard value used in cases where any of the two proteins had replicates. The boxplot compares the distribution of Jaccard similarities of replicates and pairs of different proteins. Bars represent the first and third quartiles and the *lower* and *upper* whiskers represent the lowest and highest datum still within 1.5× IQR of the lower and upper quartiles, respectively, where IQR is the interquartile range. Dashed boxes represent the whole data set, the solid-line boxes the filtered data set (Med500 score >600). The colored bars on the *top* of the heat map represent different effector domains, with the color legend shown at the *bottom*. See also Supplemental Figure S1, Supplemental Tables S1 and S4.

We examined and validated the ChIP-seq data in several ways. First, we compared the peaks obtained for pairs of proteins, and the binding motifs. Only a small proportion of pairs (∼1%) displayed strong overlap of peaks (Jaccard similarity ≥0.2), such that most (>99 out of 131) have a unique binding pattern (Fig. 1; Supplemental Table S4). This phenomenon is not due to noise or irreproducibility: When we restrict our data set to the 57 proteins with the highest quality (QC score >600, see Supplemental Methods), we still see little overlap of peaks among different proteins (4.7% with Jaccard similarity ≥0.2), whereas biological replicates in the same set are highly similar (100% with Jaccard similarity ≥0.2). Forty-two of the C2H2-ZF proteins had replicates in the ChIP-seq data (up to seven), for a total of 115 ChIP experiments with replicates. Among these, 93 (∼81%) are more similar to at least one of their replicates than to any other experiments (i.e., if we take one experiment, and rank all other experiments based on Jaccard similarity to that experiment, the top ranking experiment is from the same protein).

**Figure 2.**
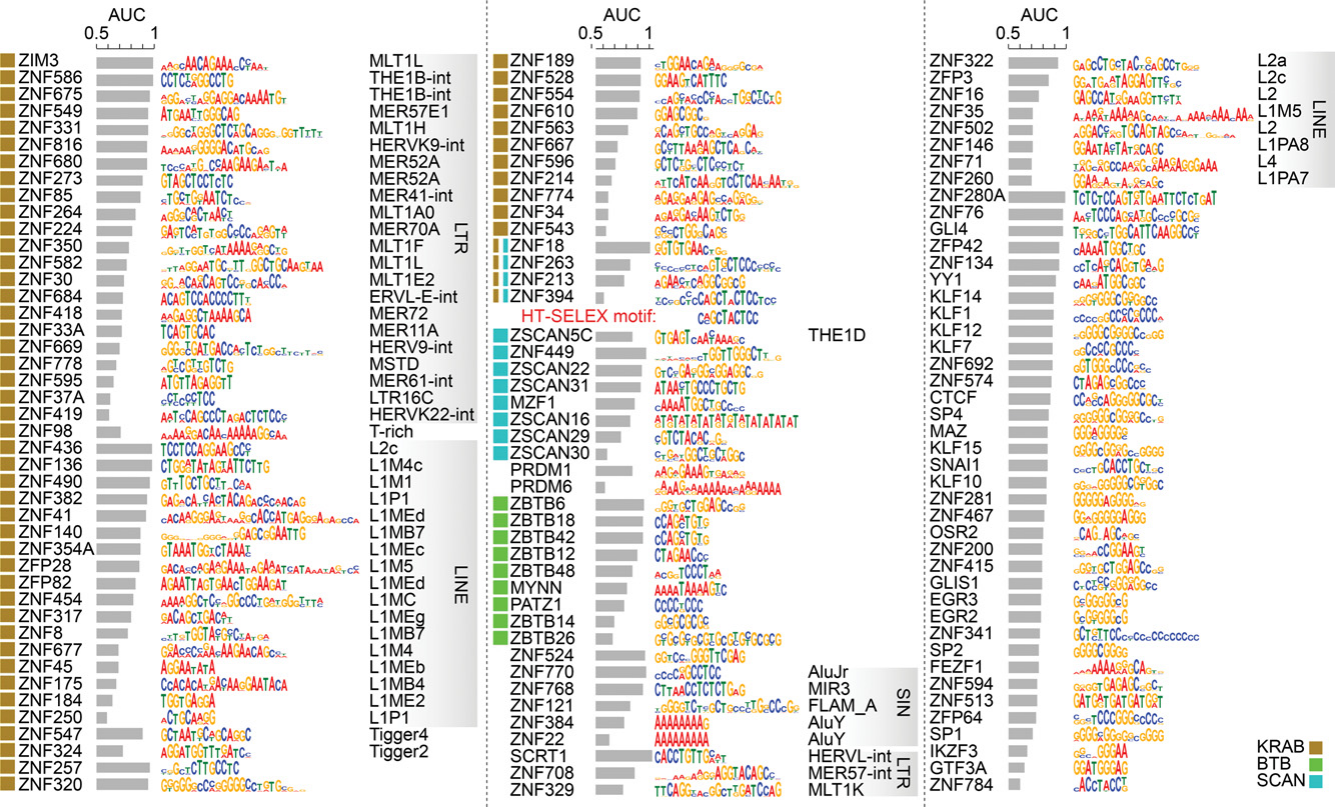
Motifs for the 131 C2H2-ZF proteins analyzed by ChIP-seq. Motifs are identified using either MEME or RCADE (see Supplemental Methods). The area under the ROC curve (AUC) of each motif for distinguishing top 500 peaks from dinucleotide-shuffled sequences is shown, along with the log odds sequence logo of the motif. For proteins whose motif-containing peaks overlap significantly with repeat elements at FDR <0.01, the repeat type with the most significant enrichment (i.e., the smallest *P*-value) is shown on the *right*. The squares on the *left* of the proteins represent effector domains. For ZNF394 both the ChIP-seq motif (*top*) and the HT-SELEX motif (*bottom*) are shown. See also Supplemental Figure S2, Supplemental Tables S2 and S3.

Consistent with their diversity in peak locations, and also with previous analyses of C2H2-ZF sequence preferences (Wolfe et al. 2000; Najafabadi et al. 2015b), the motifs derived from the ChIP-seq data are also highly diverse (Fig. 2; Supplemental Table S2). The motifs also tend to be long (average 16 bases), suggesting that they utilize a large number of C2H2-ZF domains in DNA binding. For proteins with known motifs, the motifs we identified are highly similar (Supplemental Table S2). Moreover, for ten of the remaining proteins, we found that our ChIP-seq motifs were very similar to motifs identified by HT-SELEX in an independent study (Y Yin, A Jolma, and J Taipale, in prep). We tested an additional protein by HT-SELEX (ZNF394) and again found that the motif was nearly identical (Fig. 2, center).

To ask whether binding of C2H2-ZF proteins to specific genomic loci is driven entirely by their DNA-binding activity, we scored how well the motif for each protein specifies its genomic binding sites, using AUROC analysis. A wide spectrum of AUROC values was obtained (Supplemental Fig. S2), with a median of 0.81. Peaks typically occupy <0.1% of the genome; thus, a TF with the ability to silence single-handedly all EREs that contain its recognition sequence would have to overcome any obstructive chromatin at potential binding sites and have an AUROC well over 0.99. A very small number of C2H2-ZF proteins overall appear to possess this ability, suggesting that pre-existing chromatin state and/or interacting partners play a role in genomic binding of most C2H2-ZF proteins.

**Figure 3.**
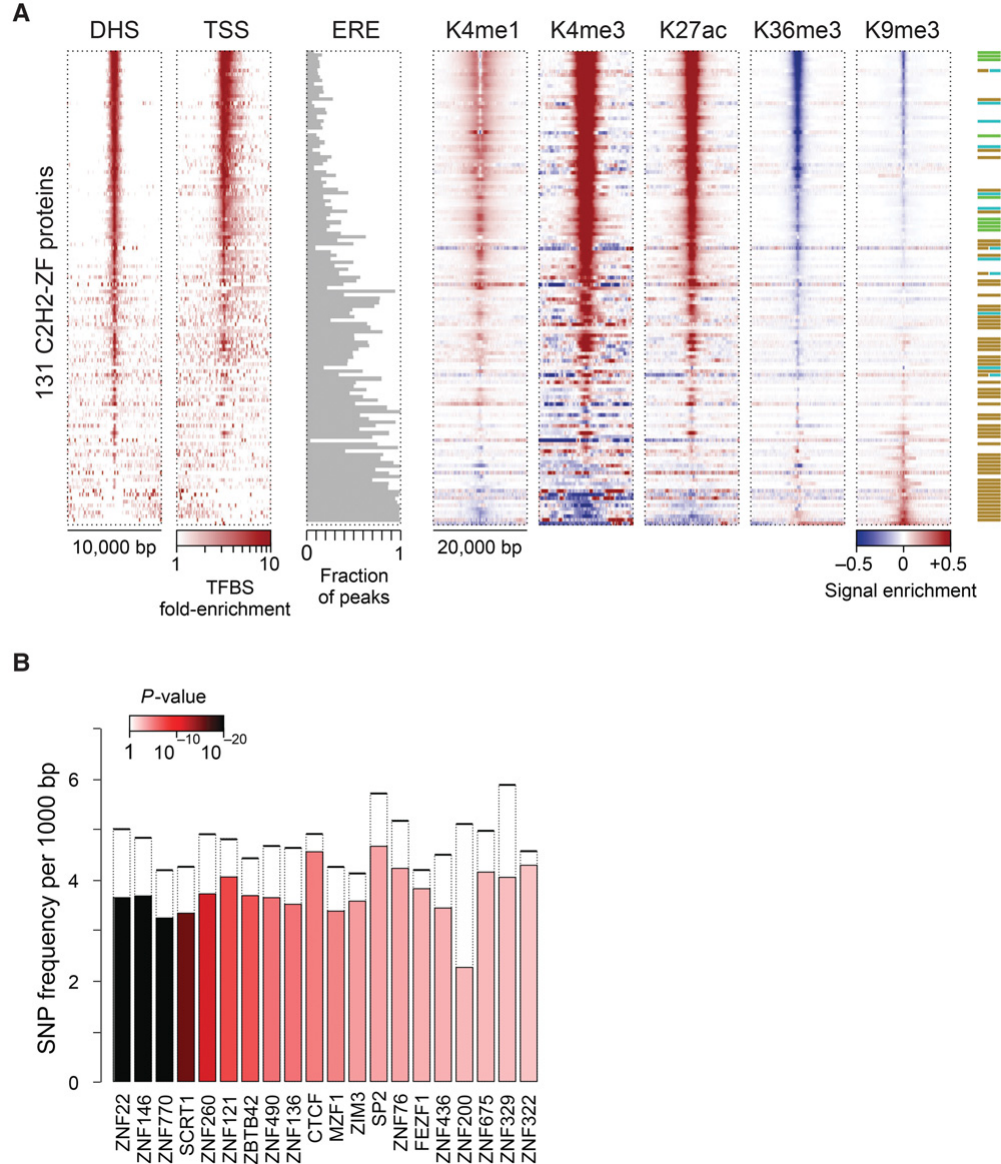
Genomic distribution of C2H2-ZF binding sites. (*A*) Enrichment of binding sites around different genomic features as well as histone marks. For DHS and TSS, the color map represents fold-enrichment of binding sites around these genomic features relative to distribution of binding sites around random genomic points. For histone marks, the color map shows base-10 logarithm of fold-change of histone mark signal around binding sites, relative to the genomic average of the signal. For EREs, the fraction of peaks that overlap any ERE instance on the genome is shown. (*B*) Distribution of common SNPs inside C2H2-ZF binding sites, compared to immediate ±20 bp flanking regions. The filled bars represent the frequency of common SNPs within motif hits in the peak regions, whereas dashed bars correspond to the SNP frequency in the ±20 bp region surrounding the motif hits. *P*-values (based on Binomial test) are shown using the color gradient. Only the proteins with significant SNP depletion at binding sites (FDR<0.025) are shown.

We next compared the peaks to chromatin modifications and other chromosomal features. We considered only peaks containing motif matches, which are more likely to be direct binding sites. Like many other TFs, C2H2-ZF binding sites are often found in DNase hypersensitive sites (DHS), particularly those close to the transcription start site (Fig. 3A; The ENCODE Project Consortium 2012; Yan et al. 2013). The majority of C2H2-ZF binding sites, however, are found outside of these regions (∼780,000 out of ∼1200,000 sites in total, or 65%). C2H2-ZF protein binding sites are also often enriched for chromatin marks that are characteristic of promoters, enhancers, or repressed regions (Fig. 3A). Most KRAB-containing C2H2-ZF proteins, however, displayed high enrichment of peaks within EREs, as previously observed (Najafabadi et al. 2015b), and much less overall association with DHS (Fig. 3A). On average, however, the motifs of the KRAB-C2H2-ZF proteins were no better at discerning peaks than the motifs of other C2H2-ZFs, on the basis of AUROC values (Supplemental Fig. S2), suggesting that their sequence preferences only partially explain how they target the EREs they bind. We observed no obvious theme for SCAN and BTB domain proteins, which were indistinguishable from other non-KRAB C2H2-ZF proteins in their association with chromatin marks and motif AUROC values, did not overlap EREs, and also did not display overlap in genomic binding sites with each other.

The correspondence between ChIP-seq peaks and other genomic features suggests that C2H2-ZF proteins are involved in gene activation, repression, regulation of EREs and possibly other functions in chromatin and transcription. We cannot infer from these analyses whether the overlapping chromatin marks are results of TF binding, or whether these TFs preferably bind to these regions. We note that most (albeit not all) of the C2H2-ZF proteins associated with H3K9me3 contain a KRAB domain, consistent with the established function of the TRIM28 cofactor, while most C2H2-ZF proteins that are strongly associated with H3K4me1/3 do not. These associations are not absolute, however: For example, 25% (15/59) of KRAB-containing C2H2-ZF proteins did not associate with H3K9me3.

Finally, we examined the relationship between motif-containing C2H2-ZF genomic binding sites, and human sequence variation. Overall, there is a depletion of common SNPs among the C2H2-ZF protein binding sites, comparing motif matches at peak centers relative to 20 bp of flanking sequence (Binomial test, *P* < 10^−71^). For 20 of the C2H2-ZF proteins, the depletion was significant on an individual basis (Fig. 3B). This observation suggests that there is evolutionary pressure on maintaining many of the C2H2-ZF binding sites, thus supporting their functional significance.

### KRAB-C2H2-ZF paralogs often bind related retroelements

**Figure 4.**
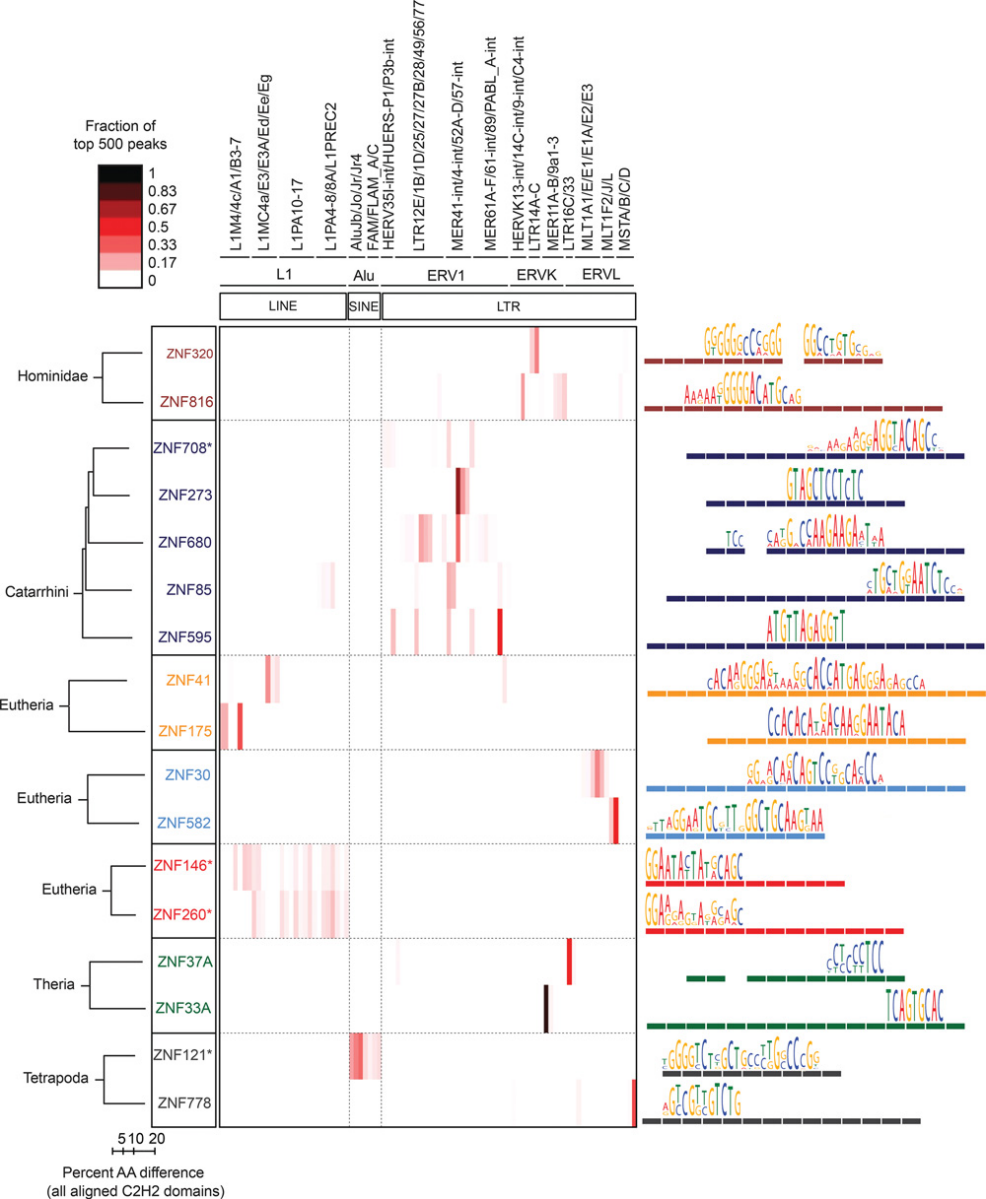
ERE binding pattern in seven groups of C2H2-ZF paralogs. The heat map (*center*) indicates the fraction of the top 500 ChIP-seq peaks overlapping each ERE (ERE classes indicated at *top*). Paralogs are grouped together in boxes (*left*) and their aligned C2H2-ZF domain structures are represented by colored rectangles (*right*) (Clustal Omega [v.1.2]) (Sievers et al. 2011). Asterisks indicate C2H2-ZF proteins that lack a KRAB domain. Taxon names at *left* indicate the most recent lineage where the paralogs share at least one homologous finger. Binding motifs (*right*) are positioned over the corresponding C2H2-ZF domains that recognize each triplet according to RCADE (Najafabadi et al. 2015a). Aligned C2H2-ZF domains of paralogs are displayed as dashed lines in the same color.

The larger number of proteins analyzed here allowed us to ask whether C2H2-ZF proteins and EREs co-evolve: Seven groups of paralogs are represented (paralog definitions are given in the Supplemental Methods; note that the specificity residues are often different among KRAB-C2H2-ZF paralogs (Emerson and Thomas 2009) and that the groups of human paralogs contain additional proteins that were not assayed here). Indeed, paralogs typically bind related classes of EREs (e.g., all bind LINE subtypes, or all bind LTR retroelements), although in most cases it is a non-overlapping subset, and in one case there is a shift between classes: ZNF778 binds MST-class ERVs, while ZNF121 binds a subset of *Alu* elements (Fig. 4). Thus, the evolution of C2H2-ZFs and EREs is not strictly linked. Moreover, even in cases where the same ERE classes are bound, the motifs often vary dramatically, consistent with changes in recognition residues (e.g., ZNF273/ZNF680). Strikingly, the DNA-binding segment of the C2H2-ZF array is also typically different between paralogs. In an extreme case, ZNF33A and ZNF37A utilize non-overlapping sets of fingers (Fig. 4, bottom). Evolutionary implications of these observations are considered in the Discussion.

### C2H2-ZF proteins often have unique PPI profiles

To gain a global picture of the molecular activities recruited by C2H2-ZF proteins, we examined PPIs by affinity purification and mass spectrometry (AP-MS). Using the same HEK293 cell lines as above, we employed the GFP-tagged C2H2-ZF proteins as baits, analyzing each protein in duplicate. The interaction scores with binding partners (preys) (either confidence estimates or peptide counts) do not form a bimodal distribution; therefore, no exact number of interactions can be specified for any protein. To highlight interactions that are most reproducible, we applied a *SAINTexpress* (Teo et al. 2014) score (AvgP) cutoff of 1, because it maximizes capture of positive controls relative to negative controls. We also excluded proteins with low variation among all samples (see Supplemental Methods and Supplemental Tables S5–S8).

**Figure 5.**
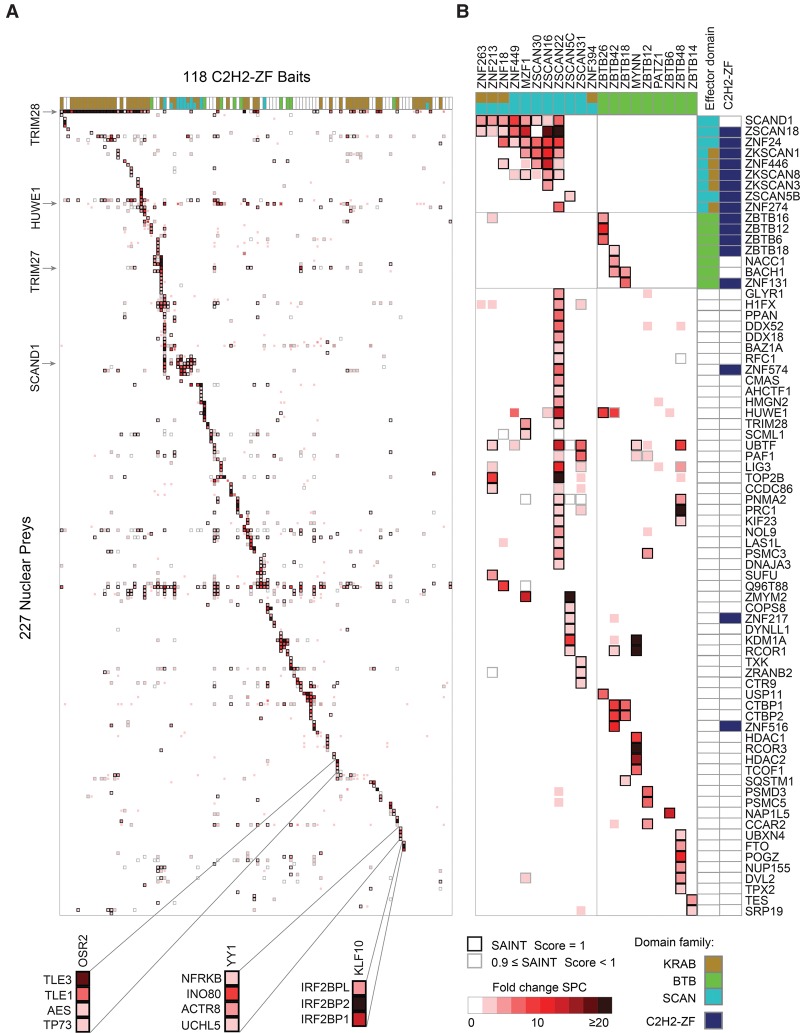
Nuclear protein interactions with C2H2-ZF proteins. AP-MS results for 118 DNA-binding C2H2-ZF proteins. (*A*) Heat map of PPIs between 118 C2H2-ZF baits and 227 nuclear prey proteins. The fill color represents the fold change spectral counts while the frame color indicates the SAINT score. Colors on *top* of the heat map represent the effector domain type of the bait proteins. Supplemental Figure S3 contains a version of the heat map with both axes fully labeled. (*B*) Detailed interactions of all SCAN- and BTB-containing bait proteins. Prey proteins are sorted by their domain type. Colors on *top* and at the *right* hand side of the heat map represent the domain types of bait and prey proteins. See also Supplemental Figures S3–S5, Supplemental Tables S5–S9.

PANTHER (Thomas et al. 2003) overrepresentation analysis of the 344 remaining preys indicates predominantly nuclear roles of the C2H2-ZF proteins in this study, consistent with the fact that all of them bound specific DNA sequences in ChIP-seq: Top scoring GO-Slim categories include ∼twofold enrichment of “nucleus” (*P* < 1.69 × 10^−5^), “RNA metabolic process” (*P* < 3.41 × 10^−6^), “DNA binding” (*P* < 0.0287); and “transcription, DNA-dependent” (*P* < 0.000883), and over fivefold enrichment of “helicase activity” (*P* < 0.0069). Figure 5A shows the PPI profiles of 118 C2H2-ZF proteins (columns) with 227 associated proteins (rows), filtered to show only nuclear proteins among the 344.

A striking aspect of the PPI data is that many of the C2H2-ZF proteins display a unique interaction profile. AP-MS data from experimental replicates were typically more similar to each other than to any of the other 117 proteins (100/118 cases) (Pearson correlation, Supplemental Fig. S7). In addition, the data reveal a high diversity of interaction partners. Many expected interactions are observed, but most were unanticipated, even for C2H2-ZF proteins with well-characterized effector domains. KRAB-containing C2H2-ZF proteins typically associate with TRIM28 (38/55 cases), but other proteins are also frequently associated (see below). The lack of significant enrichment of TRIM28 in ∼1/3 of cases was supported by the experimental replicates, and the number of spectral counts in these samples is comparable to non-KRAB proteins (Supplemental Fig. S4; Supplemental Table S9) (TRIM28 is an abundant nuclear protein and frequent low-count contaminant). In addition, there is a quantitative relationship between TRIM28 association and H3K9me3 signals (The ENCODE Project Consortium 2012) near peaks for the same protein in HEK293 cells [*r* = 0.4, (*P* < 0.0016)] (Supplemental Fig. S8). Thus, the KRAB domain may have functions other than recruitment of TRIM28.

As expected, 9 of the 11 SCAN-containing C2H2-ZF proteins we examined interact with other SCAN-containing proteins and 3 of the 9 BTB-containing C2H2-ZF proteins interact with other BTB-containing proteins (Fig. 5B). (It is possible that the lack of heterotypic interactions for some SCAN and BTB partners is explained by their absence in HEK293 cells.) In addition, in both cases, many additional specific interactions are found (Fig. 5B). Some interacting proteins are common to multiple C2H2-ZF proteins. For example, SCAND1, ZKSCAN1, and ZSCAN18 interact specifically with most of the SCAN-domain containing C2H2-ZF proteins (Fig. 5B), while the E3 ubiquitin ligase HUWE1 interacts with C2H2-ZF proteins from different subclasses, including KRAB, SCAN, BTB, and C2H2-ZF-only (Fig. 5A). Many of the interactions, however, are highly specific to one or a few C2H2-ZF proteins. The established interaction of YY1 with the INO80 complex (Cai et al. 2007) is exclusive among the 118 proteins examined, while components of the repressive DIF-1 complex (Yeung et al. 2011) interact specifically with KLF10, and Groucho-related proteins TLE1, TLE3, and AES all interact only with OSR2 (Fig. 5A). This observation is not due to thresholding effects, as the number of interacting proteins common to multiple C2H2-ZF proteins increases only slightly if confidence thresholds are lower and cytoplasmic proteins are included (Supplemental Fig. S5).

C2H2-ZF proteins also frequently associate with other C2H2-ZF proteins, beyond the expected SCAN and BTB mediated interactions (there are 35 C2H2-ZF proteins among the 227 prey proteins in Fig. 5A), but less frequently with TFs from other DBD families (only 5 of the prey proteins). Many of the interactions are between two KRAB-C2H2-ZF proteins (17 prey proteins), suggesting that KRAB may mediate oligomerization, directly or indirectly. It is also possible that some of the interactions are mediated by C2H2-ZF domains, which can interact with DNA, RNA, or protein (Brayer et al. 2008; Burdach et al. 2012).

### Many C2H2-ZF proteins interact with both transcriptional activators and repressors

**Figure 6.**
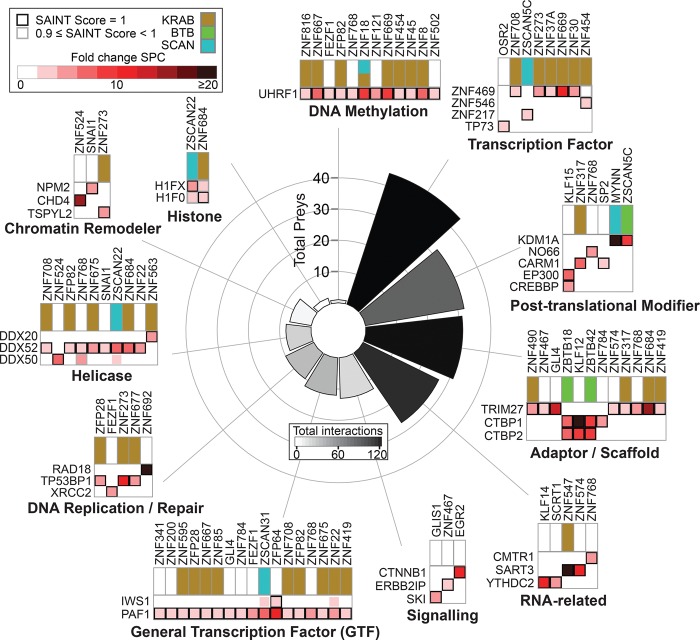
Functional overview of the C2H2-ZF protein interaction partners. All 227 nuclear prey proteins were assigned functional categories based on a literature search (see Supplemental Methods). The bar graph shows the number of individual prey proteins in each category while the color of the bars reflects the number of total interactions between bait proteins and prey proteins from each category. Example interactions for each category are shown as heat maps. See also Supplemental Figure S6 and Supplemental Table S10.

To dissect the roles of the putative cofactors recruited by each C2H2-ZF protein, we first examined categorical annotations (Gene Ontology [Gene Ontology Consortium 2015] and PANTHER [Thomas et al. 2003]) for each of the 227 associated proteins in Figure 5A. More than half (124) are associated with “regulation of gene expression,” 40 are associated with “chromosome organization,” and 20 with “histone modification,” strongly suggesting that the poorly characterized C2H2-ZF proteins are *bona fide* transcription factors that function by diverse chromatin-based mechanisms. In many cases, however, we found that the categorical annotations were based on the protein domain structure, making them relatively uninformative regarding specific molecular functions. Additionally, citations for annotations were often difficult to trace and confirm. We therefore surveyed the literature for each interacting protein, focusing on its role in regulation of transcription. We catalogued whether there is evidence that each protein is an activator or repressor, or both. We also manually classified known functions related to transcription for each protein (e.g., chromatin remodeler, protein modification, etc.). Supplemental Table S10 contains the resulting hand-curated summary of activating and repressing functions based on published literature for each interaction partner. Figure 6 provides a summary and specific examples of molecular functions.

Two overall observations emerged from this analysis. First, a wide variety of intriguing molecular functions is represented among the interacting proteins (Fig. 6; Supplemental Table S10). The largest functional categories are DNA-binding transcription factors (primarily other C2H2-ZF proteins), post-translational modifiers, and adaptor/scaffold proteins. Protein modifiers that interact with C2H2-ZF proteins also exhibit diverse activities such as histone acetylation (CREBBP/EP300) (Kalkhoven 2004), methylation (CARM1) (Chen et al. 1999), and demethylation (KDM1A, LSD1, NO66) (Shi et al. 2004; Sinha et al. 2010). The most common scaffolding protein in the interaction network is TRIM28 (Fig. 5A), followed by TRIM27 (8 interactions), CTBP1 (four interactions), and CTBP2 (three interactions), all of which have been implicated in recruitment of histone modification complexes (Bloor et al. 2005; Stankiewicz et al. 2014). These findings support a widespread role of C2H2-ZF proteins in chromatin structure and organization.

Our second overall observation is that, while 27 C2H2-ZF proteins interact with known activating cofactors (but no known repressors), and 21 interact with repressing cofactors (but no known activators), 27 interact with both. This observation, taken together with the fact that each C2H2-ZF interacts with a median of nine other proteins, suggests that multi-functionality is common among C2H2-ZF proteins. Intriguingly, this analysis includes support for the possibility that some members of the KRAB-C2H2-ZF family, which are normally thought of as repressors, act as activators of transcription (31 of the 59 KRAB-domain C2H2-ZF proteins in our data set interact with at least one effector protein that we labeled as activator of transcription). We observe, for example, an interaction between the KRAB-C2H2-ZF protein ZNF317 and CARM1 (Coactivator Associated Arginine Methyltransferase 1), and the KRAB-C2H2-ZF proteins ZFP28, ZNF273, and ZNF677 associate with the known activator TP53BP1 (Fig. 6). We note that AP-MS does not reveal stoichiometry, directness, or dependency among binding partners; thus, a complete understanding of the roles of the PPIs in C2H2-ZF-based transcription regulation will require further dissection.

To confirm that C2H2-ZF proteins can be assigned as activator or repressor on the basis of PPIs, we examined 80 of the C2H2-ZF-expressing cell lines (40 KRAB and 40 non-KRAB proteins) using RNA-seq, obtaining diverse expression profiles (Supplemental Fig. S6). Twenty-six displayed overall up- or down-regulation of genes with motif-containing ChIP-seq bound promoters (within 10 kb from TSS) for the same protein (Wilcoxon test, FDR<0.01). Of these, 24 are non-KRAB proteins that bind primarily to promoter regions, and our classification of these same 24 C2H2-ZF proteins on the basis of PPIs was strongly consistent with the up- or down-regulation observed in the RNA-seq analysis (red and blue labels, respectively, in Supplemental Figure S6).

### Multiparameter functional diversity of C2H2-ZF proteins

**Figure 7.**
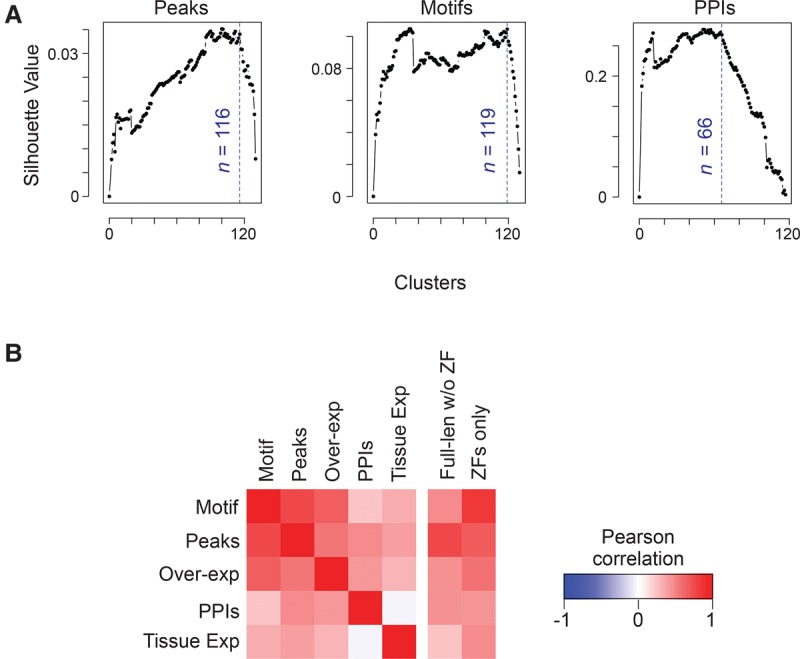
Multiparameter analysis of C2H2-ZF PPIs. (*A*) Estimated number of unique clusters of human C2H2-ZF proteins based on genomic binding sites, motifs, and PPIs. In each panel, the *x*-axis shows the number of clusters obtained by the PAM algorithm (R Core Team 2013), and the *y*-axis corresponds to the silhouette value, a measure of consistency of clustering. The blue dashed lines represent the largest number of clusters that result in 95% of the maximum silhouette value (Rousseeuw 1987), providing an estimate of the highest number of unique profiles (de Amorim and Hennig 2015) that retain high intra-cluster similarity and low inter-cluster similarity. (*B*) Correlation of functional parameters and sequence similarity among non-KRAB C2H2-ZF protein paralogs. For sequence comparison both ZF-only and full-length sequence without ZF were used. The color gradient corresponds to Pearson correlation between similarity measures. Red indicates positive correlation, i.e., when two paralogs are overall similar in one parameter, they are also similar in the other parameter, and when they have diverged in one parameter, they have also diverged in the other parameter. See also Supplemental Table S11.

Finally, we compared the diversity of genomic binding sites, DNA binding motifs, and PPIs among the 120 C2H2-ZF proteins for which we have both DNA-binding and PPI data. The data types are not directly comparable, but their diversity can be compared by examining the number of discrete groups within each data set. A related “multi-parameter” analysis of 39 *C. elegans* bHLH proteins (Grove et al. 2009) used a binary vector to simplify calculation of overlaps. To leverage the quantitative nature of our data, we instead employed a recently established framework (Wiwie et al. 2015) in which clustering quality is summarized by the “silhouette value,” which quantifies the similarity within clusters vs. similarity across clusters (Rousseeuw 1987; de Amorim and Hennig 2015). Application of this metric to different cluster numbers for genomic binding sites, motifs, and PPIs is shown in Figure 7A, which provides support that both DNA binding and PPIs exhibit a high degree of diversity, albeit with no single optimal cluster number. This conclusion is robust to variation in the clustering method (Supplemental Fig. S9) and is also consistent with manual examination of the data (e.g., Figs. 1–3, 5).

We also asked whether divergence of paralogs in one parameter is associated with divergence in another (Fig. 7B; Supplemental Table S11), or with alterations in tissue expression of the C2H2-ZF proteins (Ray et al. 2013) and with differences in the protein sequence. In general, similarity of two C2H2-ZF proteins in any of our measurements—including overexpression RNA-seq data—does not correlate strongly with similarity in tissue expression, suggesting that these parameters evolve separately. Similarity in PPIs is also poorly correlated with similarity in both measures of DNA-binding (motifs and peak overlap). In contrast, the DNA-binding measures (motifs and genomic binding sites) correlate more highly with each other, and with similarity in the C2H2-ZF domain sequence, consistent with the mechanistic linkage of these properties. Together, these observations indicate that diversification of C2H2-ZF proteins is characterized by independent and versatile evolution of DNA sequence specificity, cofactor interactions, and tissue expression.

## Discussion

In metazoans, C2H2-ZF proteins are well known for their diversification in DNA binding, and frequent association with a small number of effector domains (Stubbs et al. 2011). Surprisingly, we find that the PPIs of human C2H2-ZF proteins are nearly as diverse as their DNA binding motifs and genomic binding sites, and can vary dramatically among proteins that share the same type of effector domain. PPIs also strongly indicate that C2H2-ZF proteins do function as bona fide TFs: Most interact with at least one other protein that has an established role in regulation of chromatin or gene expression. PPIs also indicate that C2H2-ZF proteins are often multifunctional; there are many indications that the traditional classification of TFs as activators or repressors is an oversimplification (e.g., Ptashne et al. 1980; Meijsing et al. 2009; Wong and Struhl 2011) and the same appears to be true for many human C2H2-ZF proteins. Altogether, we conclude that multi-parameter evolution, previously described for bHLH proteins in *C. elegans* (Grove et al. 2009), is widespread among the largest class of human TFs.

The sequence specificity of C2H2-ZF arrays appears to evolve utilizing several attributes of their modularity, including alteration of specificity residues and domain shuffling (Stubbs et al. 2011). In addition, we find that different sets of C2H2-ZF domains are often employed among paralogs. This observation suggests that retention of unutilized C2H2-ZF modules may be beneficial over long timescales by providing a template for evolution of new DNA sequence specificities. Given a typical mammalian neutral base mutation rate of 2.2 × 10^−9^ per year (Kumar and Subramanian 2002), unselected C2H2-ZF domains should survive tens of millions of years (e.g., after 20 Mya only ∼10% of AA residues will change and ∼12% of C2H2-ZF domains would acquire a stop codon). Provided the C2H2-ZF domains are within a functional protein, stop codons would be selected against. Thus, newly duplicated C2H2-ZF domains would not require immediate selection pressure in order to be retained, and the mechanisms that produce these domains would confer long-term benefit.

Evolution of PPIs also likely involves several different mechanisms. SCAN-SCAN and BTB-BTB specificity is presumably controlled by modulation of a relatively rigid interaction surface, as these are highly structured domains, and multiple BTB-BTB structures are very similar (Stogios et al. 2005). The inter-domain contact residues vary dramatically among human SCAN and BTB proteins, consistent with the different heterodimerization partners we observe. The KRAB domain, in contrast, is believed to be largely unstructured (Mannini et al. 2006), suggesting that it may instead behave more similarly to unstructured activation domains, in which conformation is controlled by binding partners (Dyson and Wright 2005). The C2H2-ZF proteins that lack auxiliary domains are also predicted to be largely unstructured outside the C2H2-ZF domains (16% alpha helix and 6% beta sheet, overall, using HHpred [Soding et al. 2005]). The contribution of intrinsically disordered regions to PPIs is often overlooked (Oldfield and Dunker 2014); it is conceivable that the apparent excess of unstructured and poorly conserved polypeptide sequence in these proteins may serve as a template for evolution of new PPIs.

The data described here present an invaluable resource for detailed study of the large C2H2-ZF protein family, and will enable dissection of mechanisms by which they specify regulatory output at specific sites, and by which their functions evolve. The malleability of TF function in evolution is often overlooked (Lynch and Wagner 2008), and represents an obvious hurdle to comparative genomic analyses, as well as a fundamental shortcoming in the use of conservation to understand gene regulatory networks. We anticipate that our study will provide motivation for determining whether the diversity in C2H2-ZF PPIs is shared across other classes of TFs, whether it varies for orthologs across species, and the role it plays in the expansion of specific TF classes.

## Methods

### ChIP-seq

We generated HEK293 cells expressing GFP tagged C2H2-ZF proteins and performed ChIP experiments as previously described (Najafabadi et al. 2015b). We mapped ChIP-seq reads to the human genome build GRCh37 using Bowtie 2 (Langmead and Salzberg 2012). For peak calling experiment-specific background models were generated from input DNA data sets using MACS v1.4 (Zhang et al. 2008) and the Lawson-Hanson algorithm for non-negative least squares (Lawson and Hanson 1995). Peaks for individual pull-down experiments were identified using MACS v1.4 with the matching composite background reads as control. We merged summits of peaks from biological replicates that were within 50 bp of each other into a single peak, with the merged peak score being the sum of individual peak scores from the replicates, and the summit coordinate as the weighted average MACS score of the summits of the constituent peaks. A detailed description of the ChIP experiments and the data analysis parameters can be found in the Supplemental Methods.

### Motif analysis

We identified motifs using the sequence of the ±250 bp region around the top 500 peak summits for each protein, either using RCADE (Najafabadi et al. 2015a) or MEME (Bailey et al. 2009), prioritizing motifs derived from RCADE, from non-ERE peaks, and that are enriched in peak sequences. To identify motif hits inside peaks, we first identified the length of the region around peak summits that had the highest enrichment of motifs in the top 500 scoring peaks, using CentriMo (Bailey and Machanick 2012), then scanned these sequences using a motif affinity score cutoff that maximizes the enrichment of motif-containing peaks among peaks with the largest MACS scores. Details of the motif analysis are described in the Supplemental Methods.

### AP-MS procedure

We grew ∼20 million cells in two batches representing two biological replicates and harvested them 24 h following induction of protein expression with doxycycline. Cell culture conditions, sample preparation, mass spectrometry, and derivation of spectral counts were as previously described (Marcon et al. 2014). We obtained confidence scores for each putative PPI using SAINTexpress (Teo et al. 2014). Following filtering against common preys, we converted the raw sum spectral counts to odds ratios for each bait–prey interaction, by estimating the background probability of observing a peptide from each prey in the AP-MS profile of non-interacting baits. A detailed description of the AP-MS experiments and the data analysis parameters can be found in the Supplemental Methods.

### PPI analysis

PANTHER (Thomas et al. 2003) overrepresentation tests were Bonferroni corrected. For literature curation, we used a combination of PubMed, UniProt and GeneCards to assign functional tags to prey proteins and to determine the directionality of their role in transcription (activator/repressor). Publications used for the annotation are listed as PubMed IDs in Supplemental Table S4. Definitions for the functional tags can be found in the Supplemental Methods.

### RNA-seq

We grew HEK293 cells to full confluency in 6-well plates. We induced expression of C2H2-ZF proteins with doxycycline 24 h prior to harvesting. We isolated RNA using TRIzol (Thermo Fisher Scientific) as described by the manufacturer. We constructed sequencing libraries using TruSeq Stranded Total RNA Library Prep Kit with Ribo-Zero Gold or TruSeq RNA Library Preparation Kit v2. We sequenced libraries on the Illumina HiSeq 2500 to an average depth of ∼15 million 50-nucleotide reads. The data set includes 18 proteins with two or more experimental replicates (i.e., different cultures of the same cell line).

### RNA-seq data analysis

We mapped RNA-seq reads to the annotated human transcriptome using TopHat 2 (Kim et al. 2013), based on annotations from GENCODE v19 (Harrow et al. 2012). We then quantified gene-level read counts using HTSeq-count (Anders et al. 2015), and normalized them by variance-stabilizing transformation using DESeq (Anders and Huber 2010) and batch normalization.

### HT-SELEX

The HT-SELEX analysis for the ZNF394 was performed as in (Jolma et al. 2013) and the generated sequencing data was analyzed as in (Nitta et al. 2015).

## Data access

The sequencing data from this study have been submitted to the NCBI Gene Expression Omnibus (GEO; http://www.ncbi.nlm.nih.gov/geo/) under accession number GSE76496. AP-MS data have been submitted to PRIDE (https://www.ebi.ac.uk/pride/archive/) under accession number PXD003431. Sequencing reads for the HT-SELEX experiment have been submitted to European Nucleotide Archive (ENA; http://www.ebi.ac.uk/ena) under accession number PRJEB14923.

## Supplementary Material

Supplemental Material
