# Supplementary material for: Multiparameter functional diversity of human C2H2 zinc finger proteins
Source: Genome Res. 2016 Dec;26(12):1742–52. doi: 10.1101/gr.209643.116 (PMC5131825; doi:10.1101/gr.209643.116)
Supplement: Supplemental Material [file supp_26_12_1742__index.html]

Multiparameter functional diversity of human C2H2 zinc finger proteins — Supplemental Material 

# Multiparameter functional diversity of human C2H2 zinc finger proteins

## Supplemental Material

**Files in this Data Supplement:**

- Supplemental\_Methods.pdf
- Supplemental\_Figure\_S8.pdf
- Supplemental\_Figure\_S9.pdf
- Supplemental\_Figure\_S1.pdf
- Supplemental\_Figure\_S2.pdf
- Supplemental\_Figure\_S3.pdf
- Supplemental\_Figure\_S4.pdf
- Supplemental\_Figure\_S5.pdf
- Supplemental\_Figure\_S6.pdf
- Supplemental\_Table\_S2.pdf
- Supplemental\_Figure\_S7.pdf
- Supplemental\_Table\_S1.xlsx
- Supplemental\_Table\_S3.xlsx
- Supplemental\_Table\_S4.txt
- Supplemental\_Table\_S5.xlsx
- Supplemental\_Table\_S6.xlsx
- Supplemental\_Table\_S7.xlsx
- Supplemental\_Table\_S8.xlsx
- Supplemental\_Table\_S9.xlsx
- Supplemental\_Table\_S10.xlsx
- Supplemental\_Table\_S11.xlsx
