# Supplementary material for: Multiparameter functional diversity of human C2H2 zinc finger proteins
Source: Genome Res. 2016 Dec;26(12):1742–52. doi: 10.1101/gr.209643.116 (PMC5131825; doi:10.1101/gr.209643.116)
Supplement: Supplemental Material [file supp_gr.209643.116_Supplemental_Methods.pdf]

Supplemental Methods

Vector sequence. DNA sequence of the pDEST pcDNA5/FRT/TO-eGFP vector used for the ChIP-seq, AP-MS and RNA-seq experiments in this study.

GACGGATCGGGAGATCTCCCGATCCCCTATGGTGCACTCTCAGTACAATCTGCTCTGATG
CCGCATAGTTAAGCCAGTATCTGCTCCCTGCTTGTGTGTTGGAGGTCGCTGAGTAGTGCG
CGAGCAAAATTTAAGCTACAACAAGGCAAGGCTTGACCGACAATTGCATGAAGAATCTGCT
TAGGGTTAGGCGTTTTGCGCTGCTTCGCGATGTACGGGCCAGATATACGCGTTGACATTG
ATTATTGACTAGTTATTAATAGTAATCAATTACGGGGTCATTAGTTCATAGCCCATATATGGA
GTTCCGCGTTACATAACTTACGGTAAATGGCCCGCCTGGCTGACCGCCCAACGACCCCCG
CCCATTGACGTCAATAATGACGTATGTTCCCATAGTAACGCCAATAGGGACTTTCCATTGAC
GTCAATGGGTGGAGTATTTACGGTAAACTGCCCACTTGGCAGTACATCAAGTGTATCATAT
GCCAAGTACGCCCCCTATTGACGTCAATGACGGTAAATGGCCCGCCTGGCATTATGCCCA
GTACATGACCTTATGGGACTTTCCTACTTGGCAGTACATCTACGTATTAGTCATCGCTATTA
CCATGGTGATGCGGTTTTGGCAGTACATCAATGGGCGTGGATAGCGGTTTGACTCACGGG
GATTTCCAAGTCTCCACCCCATTGACGTCAATGGGAGTTTGTTTTGGCACCAAAATCAACG
GGACTTTCCAAAATGTCGTAACAACTCCGCCCCATTGACGCAAATGGGCGGTAGGCGTGT
ACGGTGGGAGGTCTATATAAGCAGAGCTCTCCCTATCAGTGATAGAGATCTCCCTATCAGT
GATAGAGATCGTCGACGAGCTCGTTTAGTGAACCGTCAGATCGCCTGGAGACGCCATCCA
CGCTGTTTTGACCTCCATAGAAGACACCGGGACCGATCCAGCCTCCGGACTCTAGCGTTT
AAACTTAAGCTTGGTACCATGGTGAGCAAGGGCGAGGAGCTGTTCACCGGGGTGGTGCCC
ATCCTGGTCGAGCTGGACGGCGACGTAAACGGCCACAAGTTCAGCGTGTCCGGCGAGGG
CGAGGGCGATGCCACCTACGGCAAGCTGACCCTGAAGTTCATCTGCACCACCGGCAAGCT
GCCCGTGCCCTGGCCCACCCTCGTGACCACCCTGACCTACGGCGTGCAGTGCTTCAGCC
GCTACCCCGACCACATGAAGCAGCACGACTTCTTCAAGTCCGCCATGCCCGAAGGCTACG
TCCAGGAGCGCACCATCTTCTTCAAGGACGACGGCAACTACAAGACCCGCGCCGAGGTGA
AGTTCGAGGGCGACACCCTGGTGAACCGCATCGAGCTGAAGGGCATCGACTTCAAGGAG
GACGGCAACATCCTGGGGCACAAGCTGGAGTACAACTACAACAGCCACAACGTCTATATC
ATGGCCGACAAGCAGAAGAACGGCATCAAGGTGAACTTCAAGATCCGCCACAACATCGAG
GACGGCAGCGTGCAGCTCGCCGACCACTACCAGCAGAACACCCCCATCGGCGACGGCCC
CGTGCTGCTGCCCGACAACCACTACCTGAGCACCCAGTCCGCCCTGAGCAAAGACCCCAA
CGAGAAGCGCGATCACATGGTCCTGCTGGAGTTCGTGACCGCCGCCGGGATCACTCTCG
GCATGGACGAGCTGTACAAGGGCGCGCCCACAAGTTTGTACAAAAAAGCTGAACGAGAAA
CGTAAAATGATATAAATATCAATATATTAAATTAGATTTTGCATAAAAAACAGACTACATAATA
CTGTAAAACACAACATATCCAGTCACTATGGCGGCCGCATTAGGCACCCCAGGCTTTACAC
TTTATGCTTCCGGCTCGTATAATGTGTGGATTTTGAGTTAGGATCCGTCGAGATTTTCAGGA
GCTAAGGAAGCTAAAATGGAGAAAAAAATCACTGGATATACCACCGTTGATATATCCCAAT
GGCATCGTAAAGAACATTTTGAGGCATTTCAGTCAGTTGCTCAATGTACCTATAACCAGACC
GTTCAGCTGGATATTACGGCCTTTTTAAAGACCGTAAAGAAAAATAAGCACAAGTTTTATCC
GGCCTTTATTCACATTCTTGCCCGCCTGATGAATGCTCATCCGGAATTCCGTATGGCAATG
AAAGACGGTGAGCTGGTGATATGGGATAGTGTTCACCCTTGTTACACCGTTTTCCATGAGC
AAACTGAAACGTTTTCATCGCTCTGGAGTGAATACCACGACGATTTCCGGCAGTTTCTACA
CATATATTCGCAAGATGTGGCGTGTTACGGTGAAAACCTGGCCTATTTCCCTAAAGGGTTT
ATTGAGAATATGTTTTTCGTCTCAGCCAATCCCTGGGTGAGTTTCACCAGTTTTGATTTAAA
CGTGGCCAATATGGACAACTTCTTCGCCCCCGTTTTCACCATGGGCAAATATTATACGCAA

GGCGACAAGGTGCTGATGCCGCTGGCGATTCAGGTTCATCATGCCGTTTGTGATGGCTTC
CATGTCGGCAGAATGCTTAATGAATTACAACAGTACTGCGATGAGTGGCAGGGCGGGGCG
TAAAGATCTGGATCCGGCTTACTAAAAGCCAGATAACAGTATGCGTATTTGCGCGCTGATT
TTTGCGGTATAAGAATATATACTGATATGTATACCCGAAGTATGTCAAAAAGAGGTATGCTA
TGAAGCAGCGTATTACAGTGACAGTTGACAGCGACAGCTATCAGTTGCTCAAGGCATATAT
GATGTCAATATCTCCGGTCTGGTAAGCACAACCATGCAGAATGAAGCCCGTCGTCTGCGT
GCCGAACGCTGGAAAGCGGAAAATCAGGAAGGGATGGCTGAGGTCGCCCGGTTTATTGAA
ATGAACGGCTCTTTTGCTGACGAGAACAGGGGCTGGTGAAATGCAGTTTAAGGTTTACACC
TATAAAAGAGAGAGCCGTTATCGTCTGTTTGTGGATGTACAGAGTGATATTATTGACACGC
CCGGGCGACGGATGGTGATCCCCCTGGCCAGTGCACGTCTGCTGTCAGATAAAGTCTCCC
GTGAACTTTACCCGGTGGTGCATATCGGGGATGAAAGCTGGCGCATGATGACCACCGATA
TGGCCAGTGTGCCGGTCTCCGTTATCGGGGAAGAAGTGGCTGATCTCAGCCACCGCGAAA
ATGACATCAAAAACGCCATTAACCTGATGTTCTGGGGAATATAAATGTCAGGCTCCCTTATA
CACAGCCAGTCTGCAGGTCGACCATAGTGACTGGATATGTTGTGTTTTACAGTATTATGTA
GTCTGTTTTTTATGCAAAATCTAATTTAATATATTGATATTTATATCATTTTACGTTTCTCGTT
CAGCTTTCTTGTACAAAGTGGTGTGACTCGAGTCTAGAGGGCCCGTTTAAACCCGCTGATC
AGCCTCGACTGTGCCTTCTAGTTGCCAGCCATCTGTTGTTTGCCCCTCCCCCGTGCCTTCC
TTGACCCTGGAAGGTGCCACTCCCACTGTCCTTTCCTAATAAAATGAGGAAATTGCATCGC
ATTGTCTGAGTAGGTGTCATTCTATTCTGGGGGGTGGGGTGGGGCAGGACAGCAAGGGG
GAGGATTGGGAAGACAATAGCAGGCATGCTGGGGATGCGGTGGGCTCTATGGCTTCTGA
GGCGGAAAGAACCAGCTGGGGCTCTAGGGGGTATCCCCACGCGCCCTGTAGCGGCGCAT
TAAGCGCGGCGGGTGTGGTGGTTACGCGCAGCGTGACCGCTACACTTGCCAGCGCCCTA
GCGCCCGCTCCTTTCGCTTTCTTCCCTTCCTTTCTCGCCACGTTCGCCGGCTTTCCCCGTC
AAGCTCTAAATCGGGGGCTCCCTTTAGGGTTCCGATTTAGTGCTTTACGGCACCTCGACCC
CAAAAAACTTGATTAGGGTGATGGTTCACGTACCTAGAAGTTCCTATTCCGAAGTTCCTATT
CTCTAGAAAGTATAGGAACTTCCTTGGCCAAAAAGCCTGAACTCACCGCGACGTCTGTCGA
GAAGTTTCTGATCGAAAAGTTCGACAGCGTCTCCGACCTGATGCAGCTCTCGGAGGGCGA
AGAATCTCGTGCTTTCAGCTTCGATGTAGGAGGGCGTGGATATGTCCTGCGGGTAAATAG
CTGCGCCGATGGTTTCTACAAAGATCGTTATGTTTATCGGCACTTTGCATCGGCCGCGCTC
CCGATTCCGGAAGTGCTTGACATTGGGGATTTCAGCGAGAGCCTGACCTATTGCATCTCCC
GCCGTGCACAGGGTGTCACGTTGCAAGACCTGCCTGAAACCGAACTGCCCGCTGTTCTGC
AGCCGGTCGCGGAGGCCATGGATGCGATCGCTGCGGCCGATCTTAGCCAGACGAGCGGG
TTCGGCCCATTCGGACCGCAAGGAATCGGTCAATACACTACATGGCGTGATTTCATATGCG
CGATTGCTGATCCCCATGTGTATCACTGGCAAACTGTGATGGACGACACCGTCAGTGCGT
CCGTCGCGCAGGCTCTCGATGAGCTGATGCTTTGGGCCGAGGACTGCCCCGAAGTCCGG
CACCTCGTGCACGCGGATTTCGGCTCCAACAATGTCCTGACGGACAATGGCCGCATAACA
GCGGTCATTGACTGGAGCGAGGCGATGTTCGGGGATTCCCAATACGAGGTCGCCAACATC
TTCTTCTGGAGGCCGTGGTTGGCTTGTATGGAGCAGCAGACGCGCTACTTCGAGCGGAGG
CATCCGGAGCTTGCAGGATCGCCGCGGCTCCGGGCGTATATGCTCCGCATTGGTCTTGAC
CAACTCTATCAGAGCTTGGTTGACGGCAATTTCGATGATGCAGCTTGGGCGCAGGGTCGA
TGCGACGCAATCGTCCGATCCGGAGCCGGGACTGTCGGGCGTACACAAATCGCCCGCAG
AAGCGCGGCCGTCTGGACCGATGGCTGTGTAGAAGTACTCGCCGATAGTGGAAACCGAC
GCCCCAGCACTCGTCCGAGGGCAAAGGAATAGCACGTACTACGAGATTTCGATTCCACCG
CCGCCTTCTATGAAAGGTTGGGCTTCGGAATCGTTTTCCGGGACGCCGGCTGGATGATCC
TCCAGCGCGGGGATCTCATGCTGGAGTTCTTCGCCCACCCCAACTTGTTTATTGCAGCTTA
TAATGGTTACAAATAAAGCAATAGCATCACAAATTTCACAAATAAAGCATTTTTTTCACTGCA
TTCTAGTTGTGGTTTGTCCAAACTCATCAATGTATCTTATCATGTCTGTATACCGTCGACCT

CTAGCTAGAGCTTGGCGTAATCATGGTCATAGCTGTTTCCTGTGTGAAATTGTTATCCGCTC
ACAATTCCACACAACATACGAGCCGGAAGCATAAAGTGTAAAGCCTGGGGTGCCTAATGA
GTGAGCTAACTCACATTAATTGCGTTGCGCTCACTGCCCGCTTTCCAGTCGGGAAACCTGT
CGTGCCAGCTGCATTAATGAATCGGCCAACGCGCGGGGAGAGGCGGTTTGCGTATTGGG
CGCTCTTCCGCTTCCTCGCTCACTGACTCGCTGCGCTCGGTCGTTCGGCTGCGGCGAGCG
GTATCAGCTCACTCAAAGGCGGTAATACGGTTATCCACAGAATCAGGGGATAACGCAGGA
AAGAACATGTGAGCAAAAGGCCAGCAAAAGGCCAGGAACCGTAAAAAGGCCGCGTTGCTG
GCGTTTTTCCATAGGCTCCGCCCCCCTGACGAGCATCACAAAAATCGACGCTCAAGTCAGA
GGTGGCGAAACCCGACAGGACTATAAAGATACCAGGCGTTTCCCCCTGGAAGCTCCCTCG
TGCGCTCTCCTGTTCCGACCCTGCCGCTTACCGGATACCTGTCCGCCTTTCTCCCTTCGG
GAAGCGTGGCGCTTTCTCATAGCTCACGCTGTAGGTATCTCAGTTCGGTGTAGGTCGTTCG
CTCCAAGCTGGGCTGTGTGCACGAACCCCCCGTTCAGCCCGACCGCTGCGCCTTATCCG
GTAACTATCGTCTTGAGTCCAACCCGGTAAGACACGACTTATCGCCACTGGCAGCAGCCA
CTGGTAACAGGATTAGCAGAGCGAGGTATGTAGGCGGTGCTACAGAGTTCTTGAAGTGGT
GGCCTAACTACGGCTACACTAGAAGAACAGTATTTGGTATCTGCGCTCTGCTGAAGCCAGT
TACCTTCGGAAAAAGAGTTGGTAGCTCTTGATCCGGCAAACAAACCACCGCTGGTAGCGG
TGGTTTTTTTGTTTGCAAGCAGCAGATTACGCGCAGAAAAAAAGGATCTCAAGAAGATCCTT
TGATCTTTTCTACGGGGTCTGACGCTCAGTGGAACGAAAACTCACGTTAAGGGATTTTGGT
CATGAGATTATCAAAAAGGATCTTCACCTAGATCCTTTTAAATTAAAAATGAAGTTTTAAATC
AATCTAAAGTATATATGAGTAAACTTGGTCTGACAGTTACCAATGCTTAATCAGTGAGGCAC
CTATCTCAGCGATCTGTCTATTTCGTTCATCCATAGTTGCCTGACTCCCCGTCGTGTAGATA
ACTACGATACGGGAGGGCTTACCATCTGGCCCCAGTGCTGCAATGATACCGCGAGACCCA
CGCTCACCGGCTCCAGATTTATCAGCAATAAACCAGCCAGCCGGAAGGGCCGAGCGCAGA
AGTGGTCCTGCAACTTTATCCGCCTCCATCCAGTCTATTAATTGTTGCCGGGAAGCTAGAG
TAAGTAGTTCGCCAGTTAATAGTTTGCGCAACGTTGTTGCCATTGCTACAGGCATCGTGGT
GTCACGCTCGTCGTTTGGTATGGCTTCATTCAGCTCCGGTTCCCAACGATCAAGGCGAGTT
ACATGATCCCCCATGTTGTGCAAAAAAGCGGTTAGCTCCTTCGGTCCTCCGATCGTTGTCA
GAAGTAAGTTGGCCGCAGTGTTATCACTCATGGTTATGGCAGCACTGCATAATTCTCTTACT
GTCATGCCATCCGTAAGATGCTTTTCTGTGACTGGTGAGTACTCAACCAAGTCATTCTGAG
AATAGTGTATGCGGCGACCGAGTTGCTCTTGCCCGGCGTCAATACGGGATAATACCGCGC
CACATAGCAGAACTTTAAAAGTGCTCATCATTGGAAAACGTTCTTCGGGGCGAAAACTCTC
AAGGATCTTACCGCTGTTGAGATCCAGTTCGATGTAACCCACTCGTGCACCCAACTGATCT
TCAGCATCTTTTACTTTCACCAGCGTTTCTGGGTGAGCAAAAACAGGAAGGCAAAATGCCG
CAAAAAAGGGAATAAGGGCGACACGGAAATGTTGAATACTCATACTCTTCCTTTTTCAATAT
TATTGAAGCATTTATCAGGGTTATTGTCTCATGAGCGGATACATATTTGAATGTATTTAGAAA
AATAAACAAATAGGGGTTCCGCGCACATTTCCCCGAAAAGTGCCACCTGACGTC

ChIP-seq procedure. We generated HEK293 cells expressing GFP tagged C2H2-ZF proteins as previously described (Najafabadi et al. 2015b). To make the expression vectors we used sequence-verified clones from the ORFeome, Harvard Plasmid and synthesized constructs. We performed chromatin immunoprecipitation as described in (Schmidt et al. 2009). In brief, we crosslinked ~20 million HEK293 cells 24 hours after induction of protein expression with

doxycycline for 10 min in 1% formaldehyde. We sonicated lysates to a DNA fragment length range of 200–300 bp using a Bioruptor (Diagenode). We then immunoprecipitated GFP-tagged transcription factors with a polyclonal anti-GFP antibody (ab290, Abcam) and Dynabeads Protein G (Invitrogen). Subsequently, we reversed crosslinks at 65 °C overnight and purified bound DNA fragments (EZ-10 Spin Column PCR Product Purification kit, Bio Basic). We constructed sequencing libraries using the Illumina TruSeq ChIP-seq kit according to the manufacturer's instructions. We sequenced libraries (single end reads) on the Illumina HiSeq 2500 to a minimum depth of 20 million 51-nucleotide reads. We sequenced two or more experimental replicates for 42 C2H2-ZF proteins (i.e. different cultures of the same cell line); 16 proteins have at least three replicates.

ChIP-seq data analysis.

Read mapping: We mapped ChIP-seq reads to the human genome build GRCh37 using Bowtie 2 (Langmead and Salzberg 2012) as previously described (Najafabadi et al. 2015b). Briefly, we trimmed the 3' ends of reads to a final length of 50 nucleotides, mapped them with the Bowtie "--very-sensitive" preset parameters, and removed duplicate reads using SAMtools (Li et al. 2009).

Peak calling: To identify regions with significant enrichment of reads, we first constructed a background model for each individual pull-down experiment by pooling together the reads from the most suitable control experiments. To do so, we first used the control experiments (i.e. input DNA material) to identify genomic regions with high read enrichment, which are most likely to represent the Sono-seq effect (Marinov et al. 2014) and other experimental artifacts and biases. We identified regions that were enriched in each control experiment using MACS v1.4 (Zhang et al. 2008) at p-value < 10^{-3}, with fragment length specified as 150 bp. We pooled enriched regions from all control experiments together and kept ±250 bp around summits of 20,000 randomly selected enriched regions. Then, for each pull-down experiment, we counted the number of reads

that overlapped these 20,000 regions, and used the Lawson-Hanson algorithm for non-negative least squares (Lawson and Hanson 1995) to identify a set of weights (i.e. regression coefficients) for the control experiments so that after pooling reads according to those weights, the composite read profile would be most similar to the read profile of the pull-down experiment across the 20,000 regions. This procedure ensured that the pooled background model would best reflect the experimental artifacts of each pull-down experiment, and also allowed us to construct high-coverage background models by pooling reads from multiple control experiments.

After construction of the experiment-specific background models, we identified peaks from individual pull-down experiments using MACS v1.4, with the matching composite background reads as control, at p-value < 10^{-3}.

Measuring the overlap of peaks from pairs of experiments: In order to quantify the overlap of ChIP-seq peaks, for each pair of experiments i and j, we first identified an optimal *p-value* cutoff for i such that peaks of i that are above that threshold would be maximally enriched among top-scoring peaks of j, and also identified a *p-value* cutoff for j that would maximize the enrichment of cutoff-passing peaks of j among top-scoring peaks of i. To do so, we first identified all peak summits from i and j that were within 500 bp of each other, and then maximized the Mann-Whitney U z-score of the difference between scores of peaks j that overlap any peak i and peaks j that do not overlap any peak i by trying various cutoffs for i. Similarly, we determined the cutoff for j by maximizing the Mann-Whitney U z-score of the difference between scores of j-overlapping peaks of the experiment i and non-overlapping peaks of the experiment i.

After determining the optimal cutoff for i and j, we calculated the Jaccard similarity coefficient as the number of peaks from the cutoff-filtered i and j sets that were within 500bp of each other (the intersection set), divided by the sum of overlapping and non-overlapping peaks from both

experiments (the union set). It should be noted that we optimized the cutoffs separately for each experiment pair.

Merging peaks from biological replicates: We merged summits of peaks from biological replicates that were within 50bp of each other into a single peak, with the merged peak score being the sum of individual peak scores from the replicates. We defined the summit coordinate of the merged peak as the weighted average of the summits of the constituent peaks, with the weight being the MACS score of those peaks. We also pooled other peaks, i.e. those with no matching peak from the replicate, together and added them to the collection of peaks, creating a single peak summit dataset for each protein. This process was designed to be robust against merging low-quality and high-quality datasets. Specifically, by calling the peaks on each dataset individually, a low-quality dataset results in low-scoring peaks that would minimally affect the scores of peaks from the higher-quality dataset when the two datasets are merged.

Motif finding: We identified motifs using the sequence of the ±250 bp region around the top 500 peak summits for each protein, either using RCADE (Najafabadi et al. 2015a) or MEME (Bailey et al. 2009). We gave priority to RCADE motifs, which are constructed by optimizing "recognition code" predictions (Najafabadi et al. 2015b) that are based on the C2H2-ZF protein sequence itself, and are therefore more likely to represent the *bona fide* direct binding site of the protein. Only in cases that the RCADE motif and the MEME motif were similar, but the MEME motif outperformed the RCADE motif considerably in terms of the AUROC value (AUROC difference >0.1), we used the MEME motif. We also gave priority to motifs that were obtained from peaks that did not overlap ERE regions: EREs have sequence similarity due to common descent, which could potentially confound motif searching algorithms (ref RCADE paper). We only included ERE regions in the motif-finding step if non-ERE peaks did not result in significant motifs. Overall, of the 131 motifs in this study, 76 are identified by RCADE using non-ERE peaks, 34 by MEME

using non-ERE peaks, 16 by RCADE using a mix of ERE and non-ERE peaks, and 5 by MEME using a mix of ERE and non-ERE peaks.

Identification of motif hits inside peaks: We scanned the sequence around each peak summit for the presence of motif hits. In order to determine the length of the sequences that would be scanned for each protein, we first identified the length of the region around peak summits that had the highest enrichment of motifs, using CentriMo (Bailey and Machanick 2012). We provided as input to CentriMo the sequences of the ±250 bp region around the top 500 peak summits for each protein. The CentriMo output included the length of the central region that has the largest enrichment of motif hits (L_{enrich}), the number of hits within this central region for the provided sequences (n_{enrich}), and the total number of hits within the provided sequences (n_{total}). We defined the scan length as $L_{scan} = L_{enrich} \times n_{total} / n_{enrich}$.

We scanned the sequences for the presence of motif hits as previously described (Najafabadi et al. 2015a). Briefly, we converted the motifs to position-specific affinity matrices (PSAMs), and gave each sequence a score reflecting the affinity of that sequence for that motif. To identify the motif score cutoff, we used a procedure similar to what is described above in the section "*Measuring the overlap of peaks from pairs of experiments*". Specifically, we determined a motif score cutoff that would maximize the enrichment of motif-containing peaks among peaks with the largest MACS scores, with the enrichment defined as the Mann-Whitney U z score of the difference of MACS scores of motif-containing peaks vs. peaks with no motif hit. Similarly, we identified a MACS score cutoff that would maximize the enrichment of cutoff-passing peaks among those with the largest motif scores. For each protein, we then used peaks that passed both the MACS score cutoff and the motif score cutoff as the set of motif-containing peaks.

AP-MS procedure. We grew ~20 million cells in two batches representing two biological replicates and harvested them 24 hours following induction of protein expression with doxycycline.

We prepared WCE (Whole Cell Extract) as previously described (Marcon et al. 2014). We immunoprecipitated GFP-tagged C2H2-ZF proteins with anti-GFP antibody (G10362, Life Technologies) overnight followed by a 2 hour incubation with Protein G Dynabeads (Invitrogen). Following 3 washes with buffer (10mM TRIS-HCl, pH7.9, 420mM NaCl, 0.1% NP-40) and two washes with no detergent buffer (10mM TRIS-HCl, pH7.9, 420mM NaCl) we eluted immunoprecipitated proteins with ammonium hydroxide and then lyophilized them. We prepared proteins for MS by in solution trypsin digestion. Briefly, we resuspended the protein pellet in 44ul of 50mM ammonium bicarbonate, reduced the sample with 100mM TCEP-HCL, alkylated it with 500mM iodoacetamide, and then digested it with 1ug of trypsin overnight at 37°C. We then desalted samples using ZipTip Pipette tips (EMD Millipore) through standard procedures. We analyzed desalted samples with an LTQ-Orbitrap Velos mass spectrometer (ThermoFisher Scientific) **.**

AP-MS data analysis. We submitted raw MS data to X! Tandem as previously described (Marcon et al. 2014), obtaining spectral counts for individual human proteins for each experiment. We then obtained confidence scores for each putative PPI using SAINTexpress (Teo et al. 2014), utilizing our two biological replicates. As negative samples for SAINT analysis we included both internal controls (GFP-only) and equivalent CRAPome (version 1.1) negative controls (Mellacheruvu et al. 2013). We chose a SAINT confidence score (AvgP) cutoff of 1 as our operating threshold for inclusion in data analyses, for the following reasons: (1) this cutoff resulted in a false positive rate of 0 (assessed using 33 CRAPome cytosolic promiscuous proteins) and a true positive rate of 0.6 (assessed using 18 literature-curated positive controls); (2) lower cutoffs introduced false-positives; (3) sparse data (such as AP-MS) is sensitive to false positives, such that a modest false-positive rate can result in a very high false discovery rate. Figures also display PPIs with SAINT AvgP 0.9-0.99, to illustrate that our overall conclusions are not greatly impacted by thresholding effects.

We rearranged the SAINTexpress output table into matrix of sum spectral counts (baits x preys). We retained sum spectral counts across two replicates in the matrix for all interactions with a prey that had at least one interaction with a confidence score equal to 1. We removed promiscuous preys that had no interactions with a z-score higher than 2.2 in the log distribution of their sum spectral counts across all baits; the value 2.2 was chosen to allow retention of TRIM28, a protein which is expected to be present in many samples. We then converted the raw sum spectral counts to odds ratios for each bait-prey interaction. To do so, for each bait-prey interaction (i,j), we first calculated the expected number of peptide counts under the null assumption that the bait and prey would not interact with each other. We did this by estimating the background probability of observing a peptide from each prey j in the AP-MS profile of non-interacting baits (i.e. baits with SAINT score < 0.5). Then, for each bait i, the expected number of peptides from prey j would be $n_e(i,j) = N(i) * p(j)$, where $n_e(i,j)$ is the expected number of peptides from prey j in the AP-MS profile of bait i, $N(i)$ is the total number of peptides in the AP-MS profile of bait i, and $p(j)$ is the background probability of observing a peptide from prey j. We then calculated the odds ratio as $OR(i,j)=[n_o(i,j)+1]/[n_e(i,j)+1]$, where $n_o(i,j)$ is the observed peptide count for prey j in the AP-MS profile of bait i (+1 is added as pseudo-count).

To focus on the role of C2H2-ZF proteins in the nucleus, we removed all preys from the matrix that did not localize to the nucleus based on GeneCards nuclear localization score (i.e. did not have a GeneCards nuclear localization score of 3 or greater). If such an annotation was not available, we applied manual assignment as nuclear or non-nuclear based on literature review. We then clustered the matrix by seriation and rearranged clusters manually to form the readable diagonal clustering in **Fig. 5**.

For **Supplemental Fig. S4** we counted the number of C2H2-ZF proteins that interacted with each prey when the confidence score cutoff to define the interaction was set at 0.8, 0.9 and 1 for all

preys that had at least one SAINT score of 1. We compared the results using the set of (all) 388 preys, and the set of 227 nuclear preys.

The PANTHER (Thomas et al. 2003) overrepresentation tests were carried out using PANTHER Version 10.0 (released 2015-05-15). The reference list included all 20814 genes in the corresponding Homo sapiens database, and p-values were Bonferroni corrected for multiple hypothesis testing.

PPI literature curation. We used a combination of literature, Uniprot and GeneCards to assign the following functional tags to all prey proteins in our AP-MS data set: Adaptor/Scaffold, Chromatin Remodeler, General Transcription Factor (GTF), RNA Related, Signaling, Transcription Factor, DNA Replication/Repair, Protein Modifier, Helicase, Histone, DNA Methylation, Other/Unknown. We assigned more than one tag to proteins that fulfilled more than one definition. The definition of the tags are as follows: *Adaptor/Scaffold*: proteins that mediate protein interactions between two other proteins. *Chromatin Remodeler*: proteins that either chaperone histones or remodel nucleosomes. *GTFs*: proteins that associate with RNA Polymerases. *RNA Related*: proteins that contain an RNA binding domain or proteins that are parts of complexes associated with RNA modification. *Signaling*: proteins that are parts of signaling pathways. *Transcription Factor*: proteins that contain a DNA binding domain. *DNA Replication/Repair*: proteins that are involved in DNA replication and/or DNA repair. *Protein Modifier*: proteins that contain a domain with catalytic activity towards other proteins. *Helicase*: proteins that can remodel nucleic acids or nucleic acid-protein complexes. *Histone*: core histones H3, H4, H2A, H2B, linker histones and histone variants. *DNA Methylation*: proteins that are involved in reading or regulating DNA methylation. *Other/Unknown*: proteins that are uncharacterized or did not fulfill the definition of any other category.

To determine the relevance of prey proteins in the regulation of transcription, we searched PubMed for a connection between the protein name and transcription. We only considered search results containing experimental evidence, i.e. knockout or knockdown experiments followed by qPCR, Microarray or RNA-seq or experiments using reporter assays. Following the results we labeled the prey proteins with a role in activation of transcription, repression of transcription or both. All publications used for the annotation are listed as PubMed IDs in **Supplemental Table S4**.

RNA-seq. We grew HEK293 cells to full confluency in 6-well plates. We induced expression of C2H2-ZF proteins with doxycycline 24 hours prior to harvesting. We isolated RNA using Trizol (Thermo Fisher Scientific) as described by the manufacturer. We constructed sequencing libraries using TruSeq Stranded Total RNA Library Prep Kit with Ribo-Zero Gold or TruSeq RNA Library Preparation Kit v2. We sequenced libraries on the Illumina HiSeq 2500 to an average depth of ~15 million 50-nucleotide reads. The dataset includes 18 proteins with two or more experimental replicates (i.e. different cultures of the same cell line).

RNA-seq data analysis. We mapped RNA-seq reads to the annotated human transcriptome using TopHat 2 (Kim et al. 2013), based on annotations from GENCODE v19 (Harrow et al. 2012). We then quantified gene-level read counts using HTSeq-count (Anders et al. 2015), and normalized them by variance-stabilizing transformation using DESeq (Anders and Huber 2010). We removed genes that on average had less than 12.8 reads per sample, with this cutoff determined based on maximizing the correlation of variance-stabilized vs. pseudocount-added values. We batch-normalized variance-stabilized counts for each gene (in the logarithmic scale) by subtracting the median value of each sequencing batch.

Definition of paralogs. To identify paralogs, we obtained sequences for zinc finger arrays of 788 human C2H2-ZF proteins and aligned them by Clustal Omega (v.1.2) using default parameters.

We selected those aligned proteins that had an identity of >65% AND indicated common ancestry on the corresponding neighbor-joining tree (belonged to the same sub-tree with no more than 20 members). From the identified proteins, we selected those that were among our 131 proteins in this study and removed the pairs that did not share any individual C2H2-ZF domains that score as homologous when considered in isolation (defined in Najafabadi *et al.*, submitted). To choose the final paralogous groups, we filtered out those protein pairs with low blast scores in a 131 against 131 proteins BLAST search (NCBI blastp, v. 2.2.31; criteria: Query coverage > 80%, Sequence identity > 50%, e-value< 1e-50).

HT-SELEX. The HT-SELEX analysis for the ZNF394 was performed as in (Jolma et al. 2013) and the generated sequencing data was analysed as in (Nitta et al. 2015).

REFERENCES

Anders S, Huber W. 2010. Differential expression analysis for sequence count data. *Genome Biol* **11**(10): R106.

Anders S, Pyl PT, Huber W. 2015. HTSeq--a Python framework to work with high-throughput sequencing data. *Bioinformatics* **31**(2): 166-169.

Bailey TL, Boden M, Buske FA, Frith M, Grant CE, Clementi L, Ren J, Li WW, Noble WS. 2009. MEME SUITE: tools for motif discovery and searching. *Nucleic Acids Res* **37**(Web Server issue): W202-208.

Bailey TL, Machanick P. 2012. Inferring direct DNA binding from ChIP-seq. *Nucleic Acids Res* **40**(17): e128.

Harrow J, Frankish A, Gonzalez JM, Tapanari E, Diekhans M, Kokocinski F, Aken BL, Barrell D, Zadissa A, Searle S et al. 2012. GENCODE: the reference human genome annotation for The ENCODE Project. *Genome Res* **22**(9): 1760-1774.

Jolma A, Yan J, Whitington T, Toivonen J, Nitta KR, Rastas P, Morgunova E, Enge M, Taipale M, Wei G et al. 2013. DNA-Binding Specificities of Human Transcription Factors. *Cell* **152**(1-2): 327-339.

Kim D, Pertea G, Trapnell C, Pimentel H, Kelley R, Salzberg SL. 2013. TopHat2: accurate alignment of transcriptomes in the presence of insertions, deletions and gene fusions. *Genome Biol* **14**(4): R36.

Langmead B, Salzberg SL. 2012. Fast gapped-read alignment with Bowtie 2. *Nat Methods* **9**(4): 357-359. Lawson CL, Hanson RJ. 1995. *Solving least squares problems*. SIAM, Philadelphia.

Li H, Handsaker B, Wysoker A, Fennell T, Ruan J, Homer N, Marth G, Abecasis G, Durbin R. 2009. The Sequence Alignment/Map format and SAMtools. *Bioinformatics* **25**(16): 2078-2079.

Marcon E, Ni Z, Pu S, Turinsky AL, Trimble SS, Olsen JB, Silverman-Gavrila R, Silverman-Gavrila L, Phanse S, Guo H et al. 2014. Human-chromatin-related protein interactions identify a demethylase complex required for chromosome segregation. *Cell Rep* **8**(1): 297-310.

Marinov GK, Kundaje A, Park PJ, Wold BJ. 2014. Large-scale quality analysis of published ChIP-seq data. *G3 (Bethesda)* **4**(2): 209-223.

Mellacheruvu D, Wright Z, Couzens AL, Lambert JP, St-Denis NA, Li T, Miteva YV, Hauri S, Sardiu ME, Low TY et al. 2013. The CRAPome: a contaminant repository for affinity purification-mass spectrometry data. *Nat Methods* **10**(8): 730-736.

Najafabadi HS, Albu M, Hughes TR. 2015a. Identification of C2H2-ZF binding preferences from ChIP-seq data using RCADE. *Bioinformatics* **31**(17): 2879-2881.

Najafabadi HS, Mnaimneh S, Schmitges FW, Garton M, Lam KN, Yang A, Albu M, Weirauch MT, Radovani E, Kim PM et al. 2015b. C2H2 zinc finger proteins greatly expand the human regulatory lexicon. *Nat Biotechnol*.

Nitta KR, Jolma A, Yin Y, Morgunova E, Kivioja T, Akhtar J, Hens K, Toivonen J, Deplancke B, Furlong EE et al. 2015. Conservation of transcription factor binding specificities across 600 million years of bilateria evolution. *eLife* **4**.

Schmidt D, Wilson MD, Spyrou C, Brown GD, Hadfield J, Odom DT. 2009. ChIP-seq: using high-throughput sequencing to discover protein-DNA interactions. *Methods* **48**(3): 240-248.

Teo G, Liu G, Zhang J, Nesvizhskii AI, Gingras AC, Choi H. 2014. SAINTexpress: improvements and additional features in Significance Analysis of INTeractome software. *Journal of proteomics* **100**: 37-43.

Thomas PD, Campbell MJ, Kejariwal A, Mi H, Karlak B, Daverman R, Diemer K, Muruganujan A, Narechania A. 2003. PANTHER: a library of protein families and subfamilies indexed by function. *Genome Res* **13**(9): 2129-2141.

Zhang Y, Liu T, Meyer CA, Eeckhoute J, Johnson DS, Bernstein BE, Nusbaum C, Myers RM, Brown M, Li W et al. 2008. Model-based analysis of ChIP-Seq (MACS). *Genome Biol* **9**(9): R137.
