# Supplementary material for: Multiparameter functional diversity of human C2H2 zinc finger proteins
Source: Genome Res. 2016 Dec;26(12):1742–52. doi: 10.1101/gr.209643.116 (PMC5131825; doi:10.1101/gr.209643.116)
Supplement: Supplemental Material [file supp_gr.209643.116_Supplemental_Figure_S8.pdf]

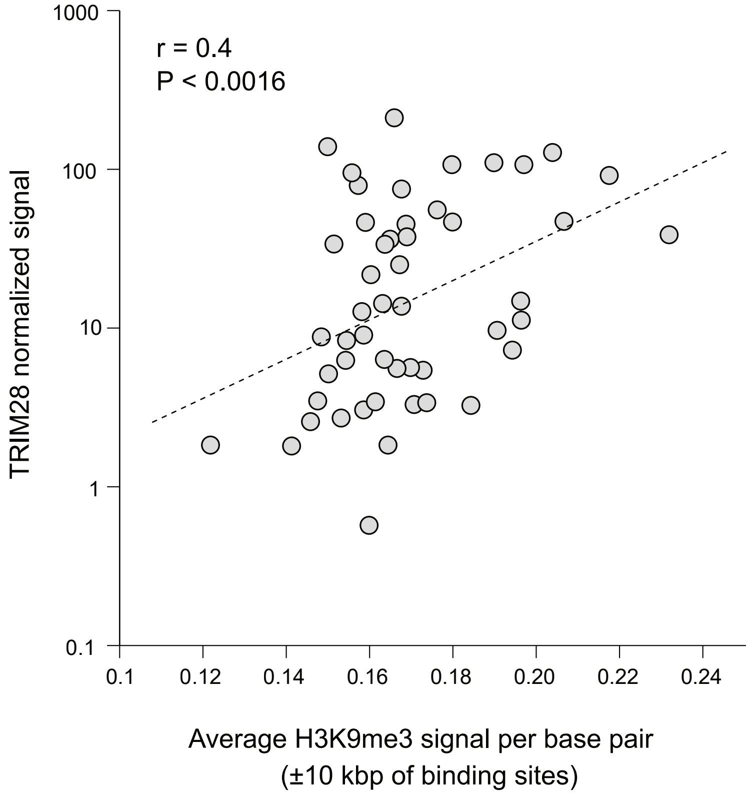

Supplemental Figure S8 (related to Figure 5): Correlation between TRIM28 association and H3K9me3 signals. Scatterplot of TRIM28 signal vs. H3K9me3 signal for the 50 KRAB proteins that were studied by AP-MS and ChIP-seq. The Pearson correlation is calculated using the log-transformed TRIM28 signal.
