# Supplementary material for: Multiparameter functional diversity of human C2H2 zinc finger proteins
Source: Genome Res. 2016 Dec;26(12):1742–52. doi: 10.1101/gr.209643.116 (PMC5131825; doi:10.1101/gr.209643.116)
Supplement: Supplemental Material [file supp_gr.209643.116_Supplemental_Figure_S9.pdf]

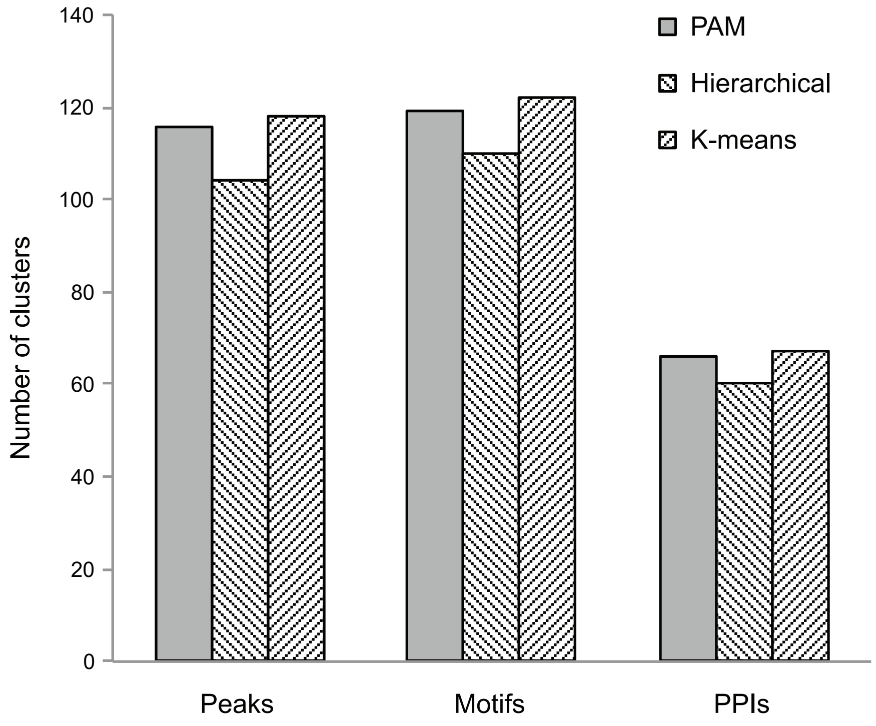

Supplemental Figure S9 (related to Figure 7): Number of clusters that are obtained for different parameters of C2H2-ZF proteins using different clustering methods. In each case, the largest number of clusters that results in 95% of the maximum silhouette value is shown.
