# Supplementary material for: Multiparameter functional diversity of human C2H2 zinc finger proteins
Source: Genome Res. 2016 Dec;26(12):1742–52. doi: 10.1101/gr.209643.116 (PMC5131825; doi:10.1101/gr.209643.116)
Supplement: Supplemental Material [file supp_gr.209643.116_Supplemental_Figure_S1.pdf]

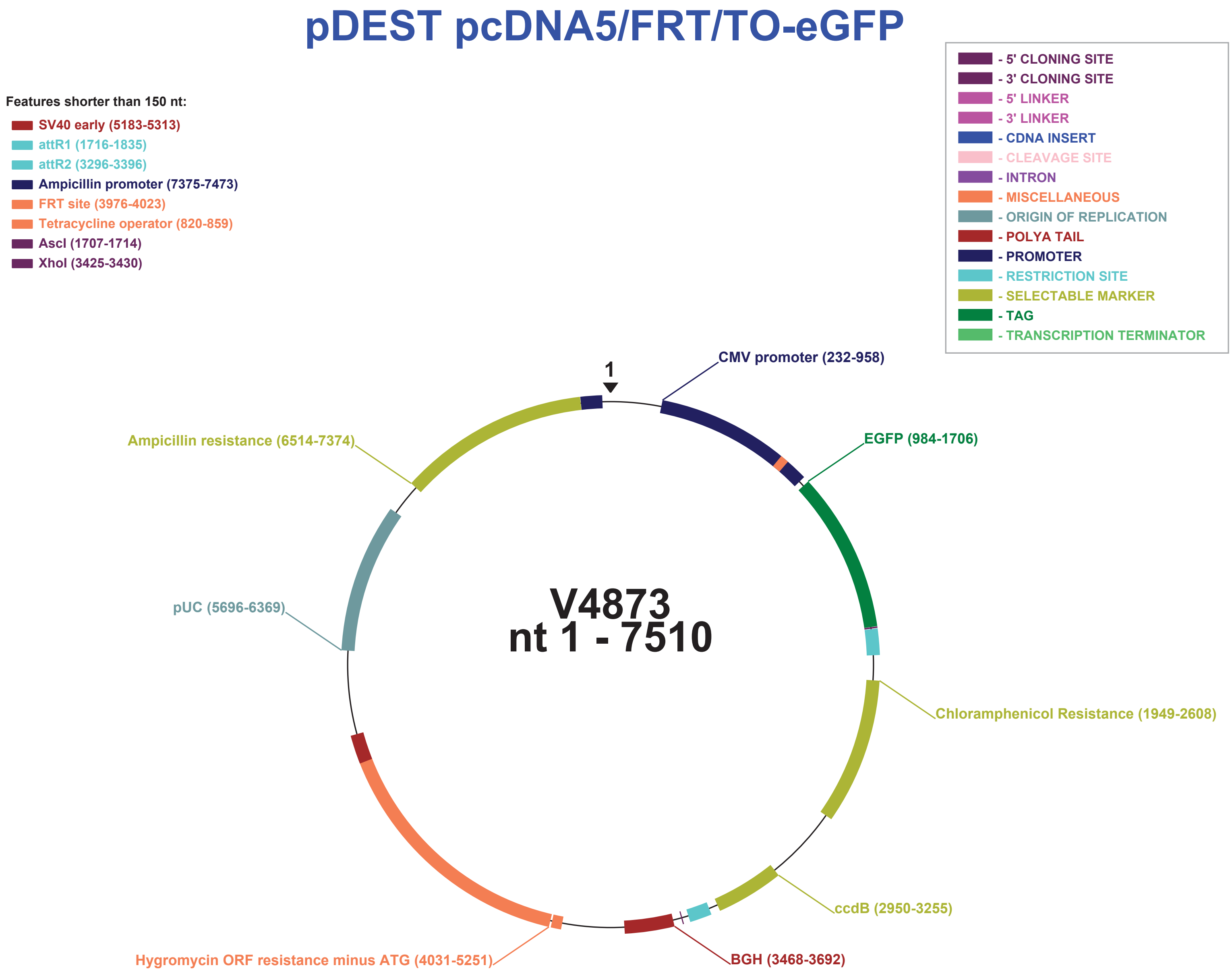

Supplemental Figure S1 (related to Figure 1): Vector map. Map of the pDEST pcDNA5/FRT/-TO-eGFP vector used for the ChIP-seq, AP-MS and RNA-seq experiments in this study.
