# Supplementary material for: Multiparameter functional diversity of human C2H2 zinc finger proteins
Source: Genome Res. 2016 Dec;26(12):1742–52. doi: 10.1101/gr.209643.116 (PMC5131825; doi:10.1101/gr.209643.116)
Supplement: Supplemental Material [file supp_gr.209643.116_Supplemental_Figure_S2.pdf]

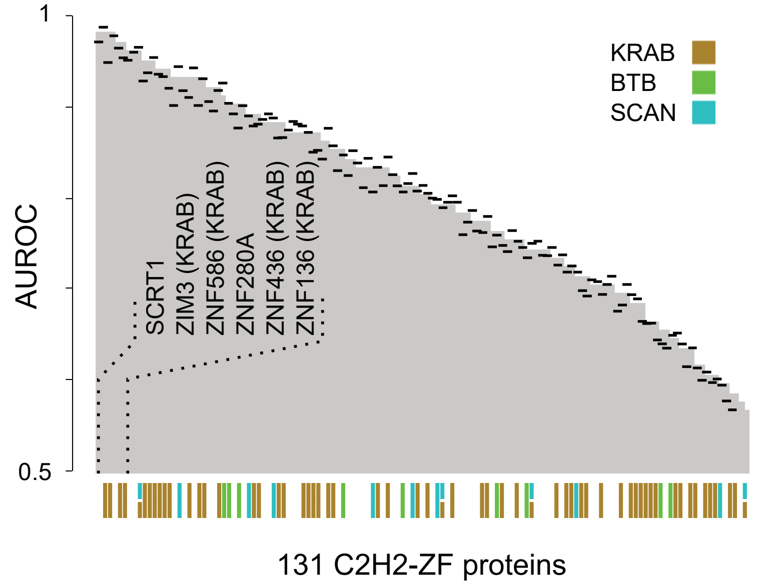

Supplemental Figure S2 (related to Figure 2). Distribution of AUROC values for 131 C2H2-ZF protein DNA-binding motifs, tested on ChIP-seq peaks. The AUROCs are calculated as described (Najafabadi et al., 2015b), comparing the top 500 peaks of each protein to dinucleotide-shuffled sequences (grey) or 500 randomly-selected genomic regions filtered to contain a matching distribution of length and dinucleotides, and to be composed of non-peak, non-ERE regions of the genome (black dashes). Effector domains are shown below; proteins with AUROC>0.98 are indicated with names.
