# Supplementary figures and images for: Multiparameter functional diversity of human C2H2 zinc finger proteins
Source: Genome Res. 2016 Dec;26(12):1742–52. doi: 10.1101/gr.209643.116 (PMC5131825; doi:10.1101/gr.209643.116)

## 118 C2H2 Baits

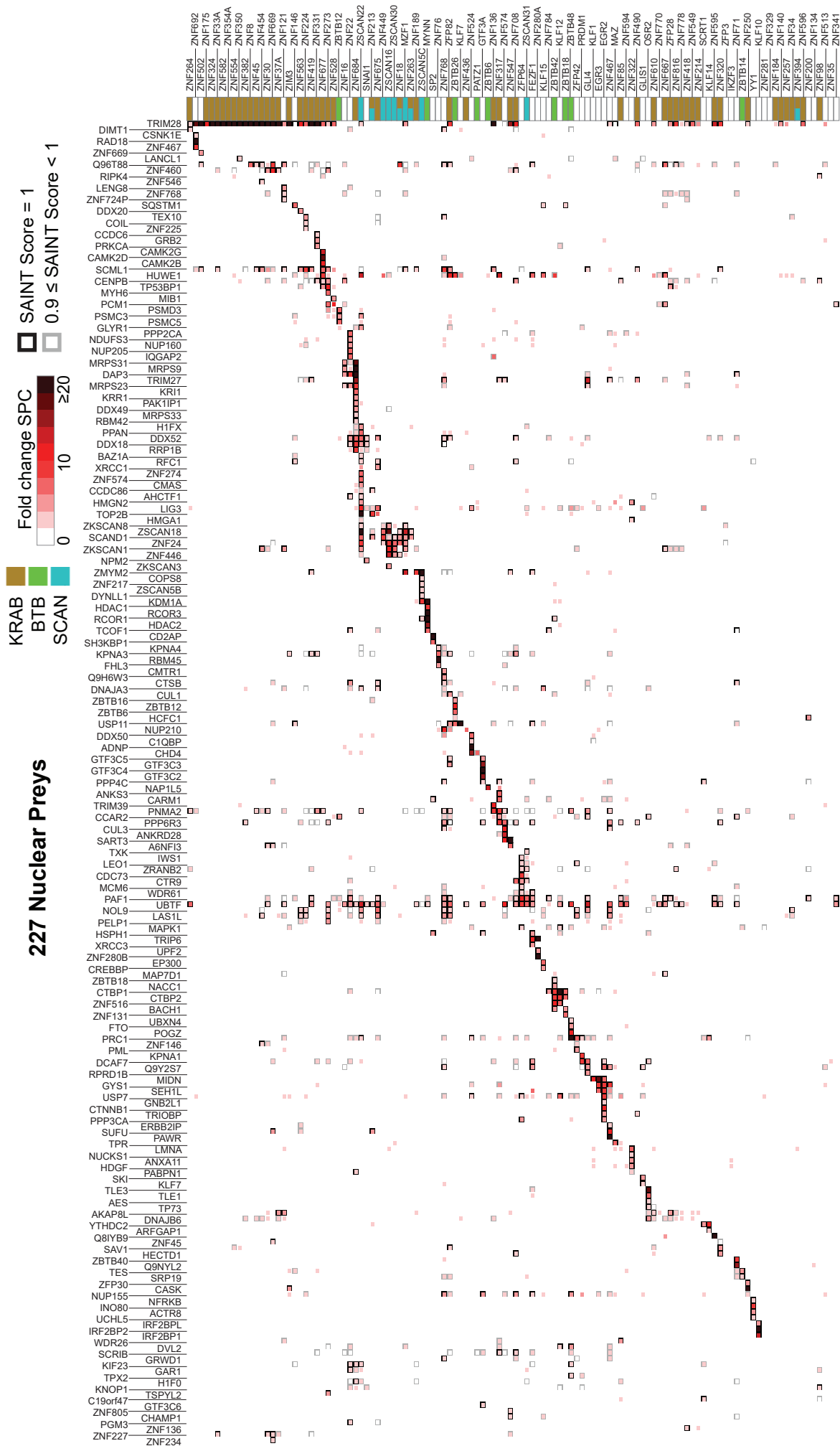

**Supplemental Figure S3**  
(related to Figure 5).  
Fully labeled version of  
Figure 5A.

Supplement: Supplemental Material [file supp_gr.209643.116_Supplemental_Figure_S3.pdf]
