# Supplementary material for: Multiparameter functional diversity of human C2H2 zinc finger proteins
Source: Genome Res. 2016 Dec;26(12):1742–52. doi: 10.1101/gr.209643.116 (PMC5131825; doi:10.1101/gr.209643.116)
Supplement: Supplemental Material [file supp_gr.209643.116_Supplemental_Figure_S4.pdf]

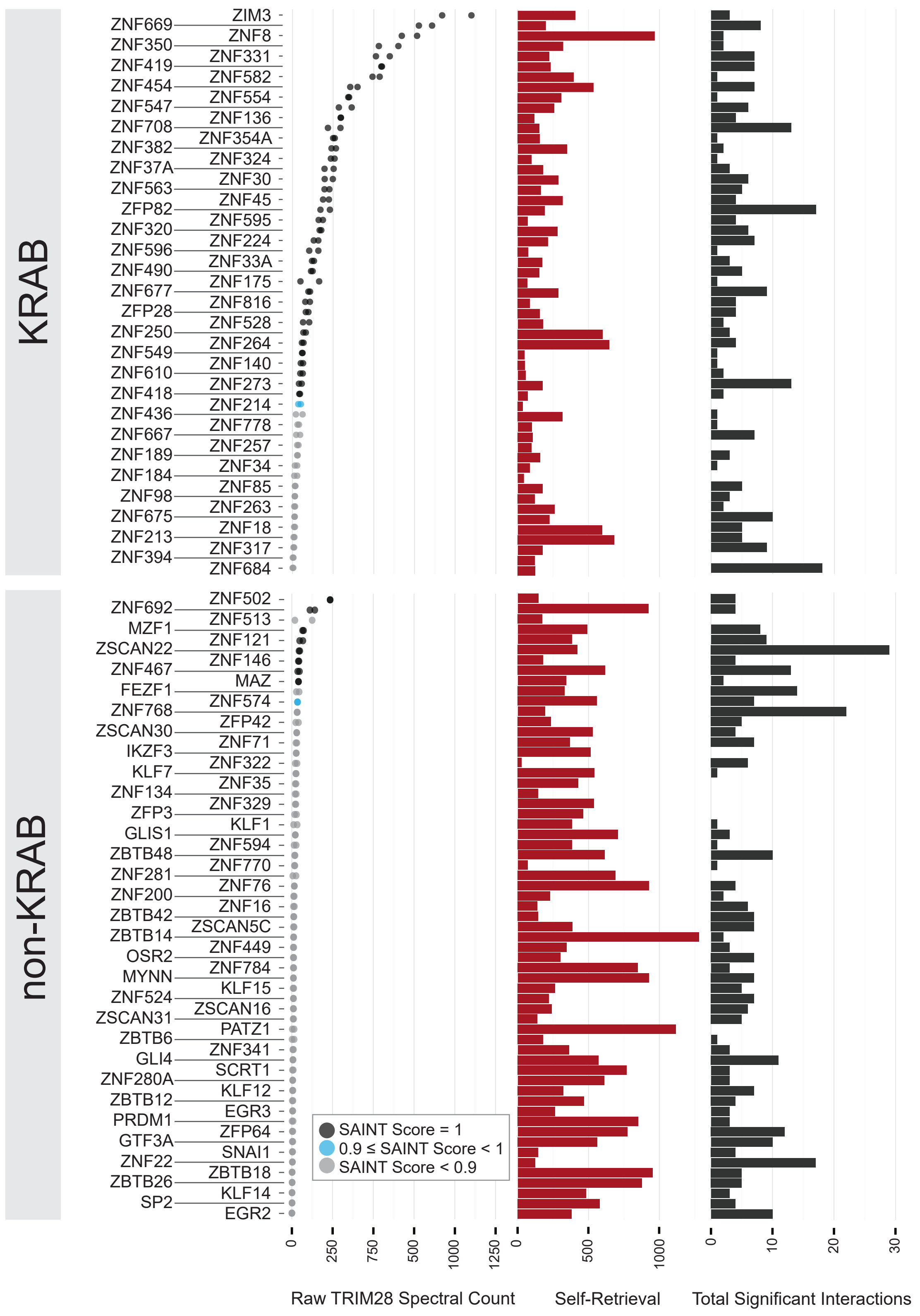

Supplemental Figure S4: (related to Figure 5). Reproducibility of TRIM28 interactions detected by AP-MS. Spectral count for TRIM28 for all 118 baits, both biological replicate purifications are indicated by dots (left). Baits are ordered by the sum spectral count for TRIM28 across both replicates. Red bars (middle) indicate self-retrieval (the bait's own spectral count in its own purification) summed across both replicates. Gray bars (right) show the number of significant interacting proteins for each bait (SAINT Score of 1).
