# Supplementary material for: Multiparameter functional diversity of human C2H2 zinc finger proteins
Source: Genome Res. 2016 Dec;26(12):1742–52. doi: 10.1101/gr.209643.116 (PMC5131825; doi:10.1101/gr.209643.116)
Supplement: Supplemental Material [file supp_gr.209643.116_Supplemental_Figure_S5.pdf]

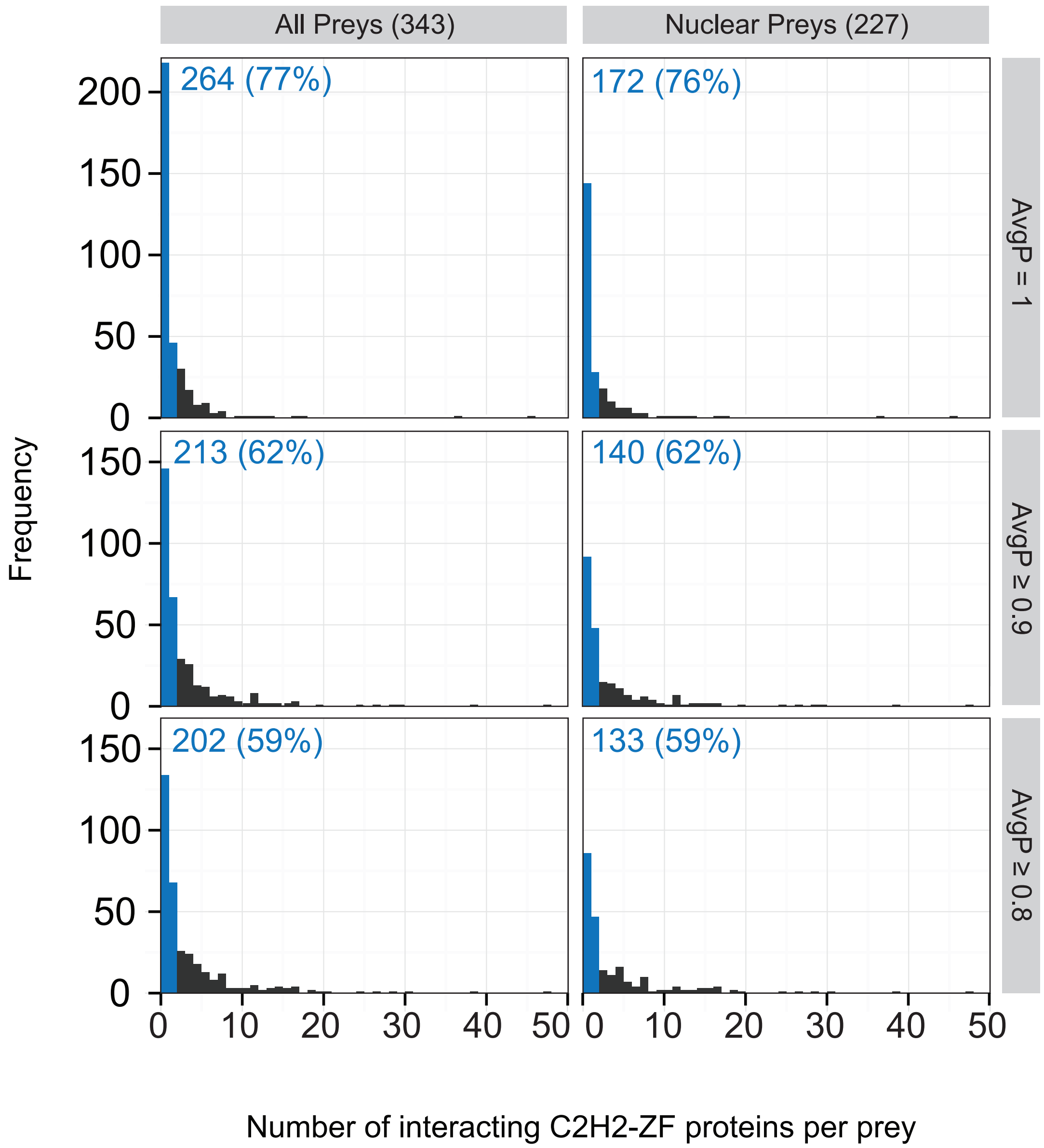

Supplemental Figure S5: (related to Figure 5). Shared interactions between C2H2-ZF proteins. The histograms display the distribution of number of C2H2-ZF proteins that purified with each prey at a given confidence score cutoff to define the interaction (including/excluding cytoplasmic preys). The results presented in Figure 5 correspond to the plot with nuclear preys and AvgP ≥ 0.9 (middle right). Blue columns indicate the total number of preys that interact with only one or two of the 118 C2H2-ZFPs examined by AP-MS.
