# Supplementary material for: Multiparameter functional diversity of human C2H2 zinc finger proteins
Source: Genome Res. 2016 Dec;26(12):1742–52. doi: 10.1101/gr.209643.116 (PMC5131825; doi:10.1101/gr.209643.116)
Supplement: Supplemental Material [file supp_gr.209643.116_Supplemental_Figure_S6.pdf]

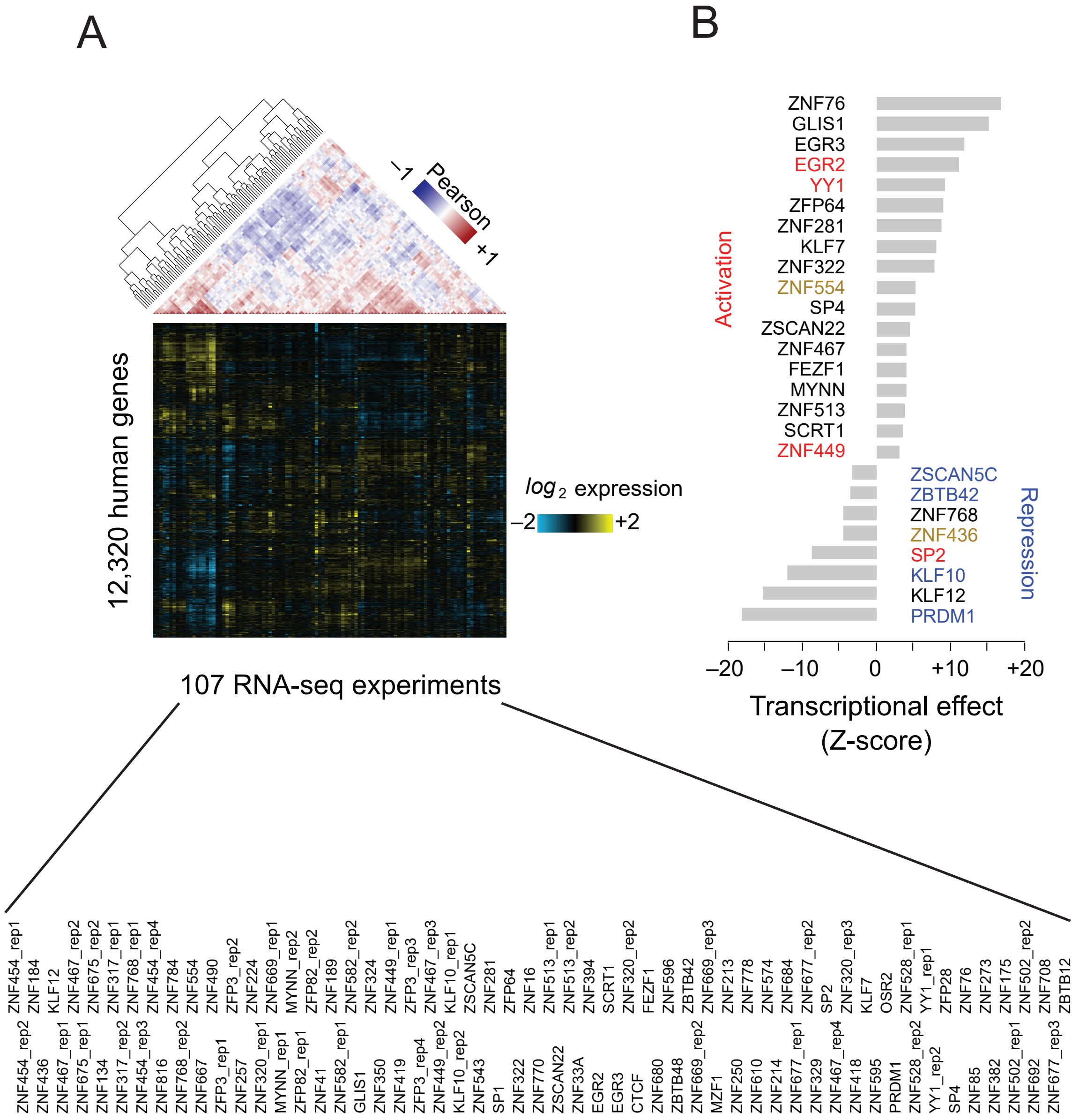

Supplemental Figure S6 (related to Figure 6): RNA-seq analysis of C2H2-ZFprotein over-expression cell lines. (A) Heatmap of expression profiles obtained from over-expression of 80 C2H2-ZF proteins in 107 independent HEK293 cell lines. (B) Mann-Whitney U Z-score for up-/down-regulation of TF target genes after over-expression of each TF. Target genes are defined as those with at least one TF binding site within 10kb of their TSS's. The Z-score for each TF indicates whether the distribution of log2 fold-change of expression for its target genes is significantly higher (positive Z-score) or lower (negative Z-score) than the distribution of log2 fold-change values of the rest of the genes (Mann-Whitney U test Z-score). Red and blue labels correspond to non-KRAB proteins that exclusively interact with co-¬activators or co-repressors, respectively. KRAB proteins are shown in brown. (C) Over-expression of each C2H2-ZF protein results in specific and reproducible remodeling of the transcriptome. In each panel, similarity of expression profiles from over-expression of different proteins is compared to that of same proteins (i.e. biological replicates). Bars represent the first and third quartiles and the lower and upper whiskers represent the lowest and highest datum still within 1.5x IQR of the lower and upper quartiles, respectively, where IQR is the interquartile range.
