# Supplementary material for: Multiparameter functional diversity of human C2H2 zinc finger proteins
Source: Genome Res. 2016 Dec;26(12):1742–52. doi: 10.1101/gr.209643.116 (PMC5131825; doi:10.1101/gr.209643.116)
Supplement: Supplemental Material [file supp_gr.209643.116_Supplemental_Table_S2.pdf]

Supplemental Table S2 (related to Figure 2): Proteins analyzed in this study, and motifs obtained using RCADE (Najafabadi et al., 2015b). Table of all C2H2-ZF proteins used in this study, including information about auxiliary domains, number of zinc finger domains and evolutionary age (in million years). Motif logos generated using RCADE are displayed for all proteins and established motifs if available. The symbol "*" marks C2H2-ZF proteins previously published in Najafabadi et al., 2015a.

Symbol	UniProt ID	Effector Domains	Number of ZF domains	Evolutionary age (Mya)	ChIP-seq motif/ established motif
FEZF1	A0PJY2	none	6	936	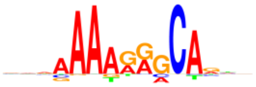
ZNF320	A2RRD8	KRAB	11	105	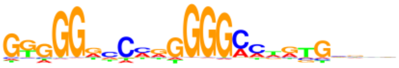
ZSCAN5C	A6NGD5	SCAN	5	43.1	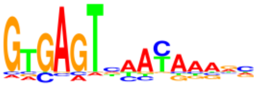
ZNF98	A6NK75	KRAB	13	97.5	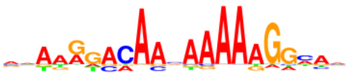
ZBTB42	B2RXF5	BTB	4	429.6	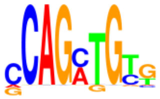
ZNF213	O14771	SCAN,KRAB	5	179.2	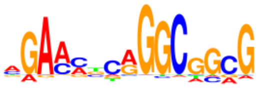
ZNF263	O14978	SCAN,KRAB	9	320.5	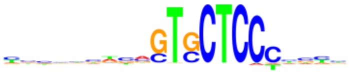 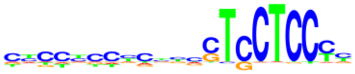 (PMID 22955619)
ZNF264*	O43296	KRAB	13	105	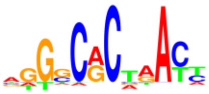
ZBTB14	O43829	BTB	5	429.6	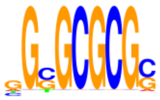
ZNF354A	O60765	KRAB	13	105	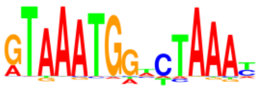
ZNF324	O75467	KRAB	9	105	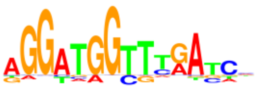

PRDM1	O75626	SET	5	847	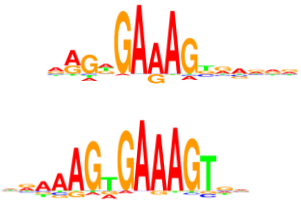 (PMID 22955619)
ZNF189*	O75820	KRAB	16	105	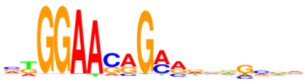
KLF7	O75840	none	3	936	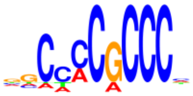
SNAI1	O95863	none	4	936	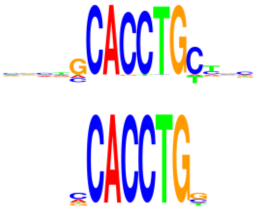 (PMID 23175603)
SP1	P08047	none	3	936	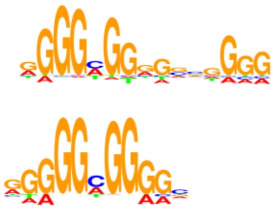 (PMID 24194598)
ZSCAN22*	P10073	SCAN	8	105	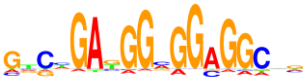
ZBTB48	P10074	BTB	11	355.7	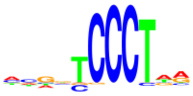
GLI4	P10075	none	7	163.7	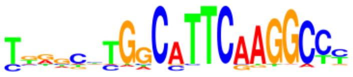
EGR2	P11161	none	3	936	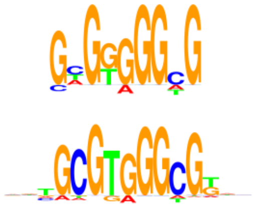 (PMID 23332764)

ZNF35*	P13682	none	11	105	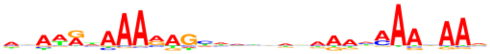
ZNF250*	P15622	KRAB	13	105	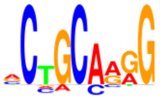
ZNF708	P17019	none	15	320.5	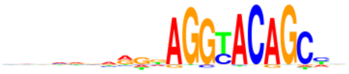
ZNF16*	P17020	none	15	163.7	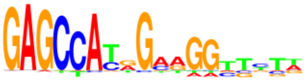
ZNF18	P17022	SCAN,KRAB	5	105	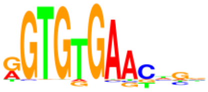
ZNF22	P17026	none	5	163.7	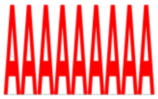
ZNF37A	P17032	KRAB	10	320.5	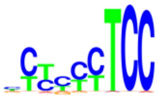
ZNF30	P17039	KRAB	16	105	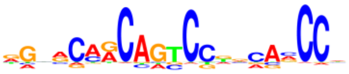
ZNF8	P17098	KRAB	7	105	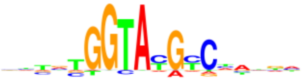
YY1*	P25490	none	4	936	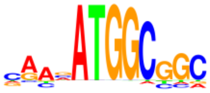<br>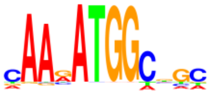<br>(PMID 24194598)
MZF1*	P28698	SCAN	13	105	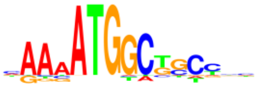<br>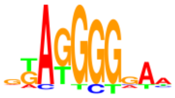<br>(PMID 24194598)
ZNF76	P36508	none	7	936	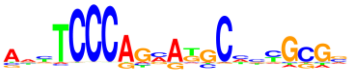

CTCF*	P49711	none	11	429.6	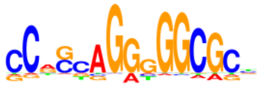 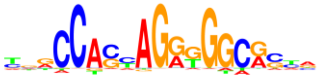 (PMID 24194598)
ZNF41*	P51814	KRAB	17	105	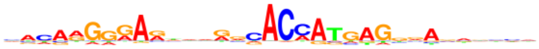
ZNF136*	P52737	KRAB	13	105	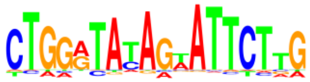
ZNF140	P52738	KRAB	10	105	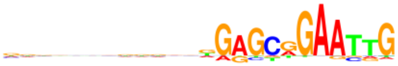
ZNF134	P52741	none	10	97.5	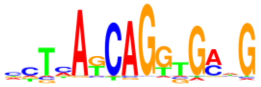
MAZ	P56270	none	5	429.6	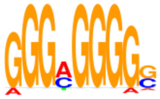 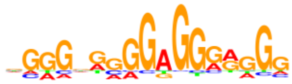 (PMID 23175603)
ZNF121	P58317	none	9	90.9	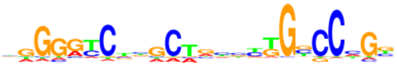
ZNF280A	P59817	none	5	0	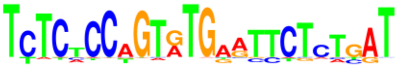
ZNF200*	P98182	none	5	105	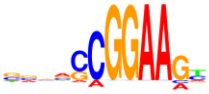
SP2	Q02086	none	3	936	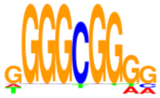 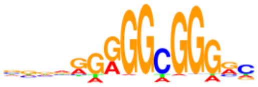 (PMID 24194598)
ZNF45*	Q02386	KRAB	15	105	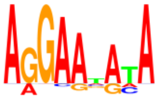

SP4	Q02446	none	3	936	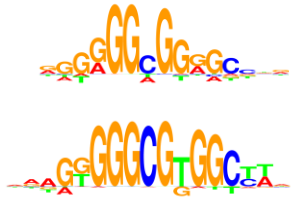 (PMID 23332764)
ZNF85	Q03923	KRAB	15	105	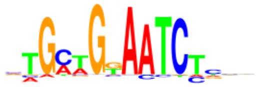
ZNF33A*	Q06730	KRAB	16	105	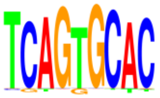
EGR3	Q06889	none	3	936	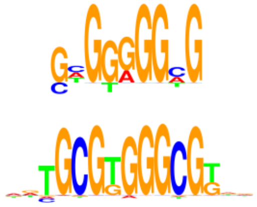 (PMID 23175603)
ZNF543	Q08ER8	KRAB	13	105	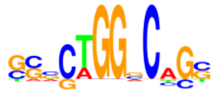
ZNF415*	Q09FC8	none	11	6.6	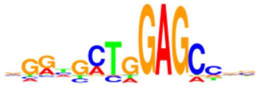
ZNF816	Q0VGE8	KRAB	15	105	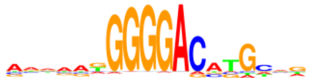
KLF10*	Q13118	none	3	936	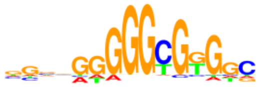
KLF1	Q13351	none	3	936	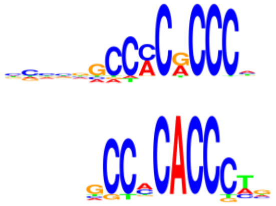 (PMID 23175603)
ZNF273*	Q14593	KRAB	10	97.5	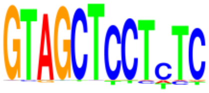
ZNF146	Q15072	none	10	105	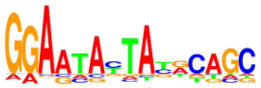

ZBTB6	Q15916	BTB	4	320.5	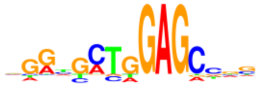 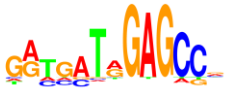 (PMID 16381825)
ZNF528*	Q3MIS6	KRAB	15	105	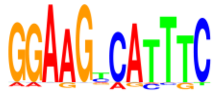
ZNF260	Q3ZCT1	none	13	105	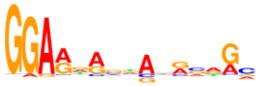
ZNF394	Q53GI3	SCAN,KRAB	6	163.7	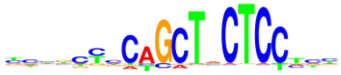
ZNF667	Q5HYK9	KRAB	14	105	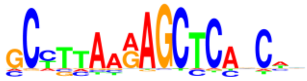
ZNF684*	Q5T5D7	KRAB	8	105	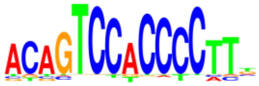
ZNF770	Q6IQ21	none	10	105	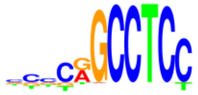
ZNF774	Q6NX45	KRAB	12	163.7	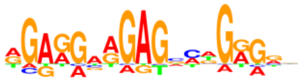
ZNF549	Q6P9A3	KRAB	13	105	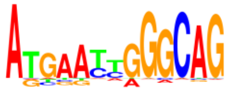
ZNF449	Q6P9G9	SCAN	7	105	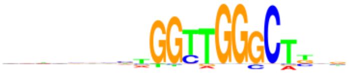
ZNF322*	Q6U7Q0	none	8	105	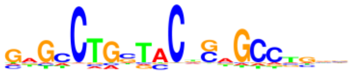
ZNF574*	Q6ZN55	none	19	163.7	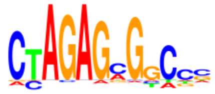
ZNF467*	Q7Z7K2	none	12	163.7	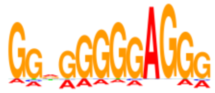

ZNF554	Q86TJ5	KRAB	7	105	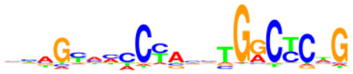
ZNF329	Q86UD4	none	12	105	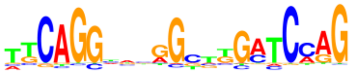
ZSCAN30	Q86W11	SCAN	7	163.7	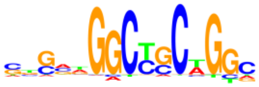
ZNF677	Q86XU0	KRAB	11	105	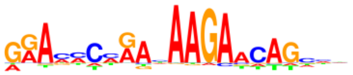
ZNF547	Q8IVP9	KRAB	10	105	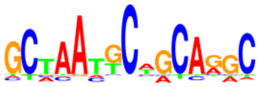
ZSCAN29	Q8IWY8	SCAN	6	179.2	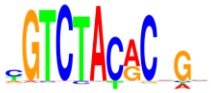
ZNF595	Q8IYB9	KRAB	17	105	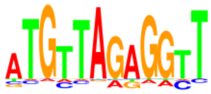
ZNF34	Q8IZ26	KRAB	12	105	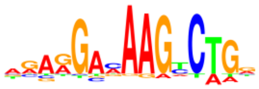
ZFP82	Q8N141	KRAB	12	105	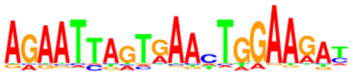
OSR2	Q8N2R0	none	5	847	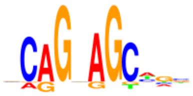
ZNF513	Q8N8E2	none	8	320.5	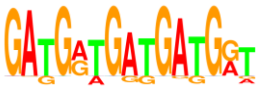
ZNF454*	Q8N9F8	KRAB	12	105	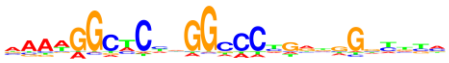
ZNF610	Q8N9Z0	KRAB	8	105	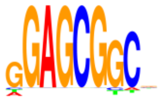

GLIS1	Q8NBF1	none	4	936	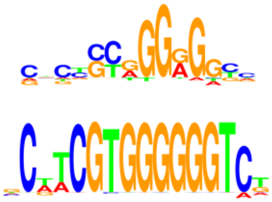 (PMID 23332764)
ZNF784	Q8NCA9	none	6	105	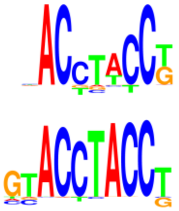 (PMID 23332764)
ZNF680	Q8NEM1	KRAB	12	97.5	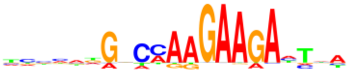
ZFP28	Q8NHY6	KRAB,KRAB	14	105	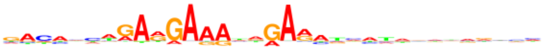
ZNF563	Q8TA94	KRAB	11	43.1	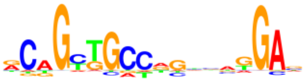
ZNF502	Q8TBZ5	none	14	105	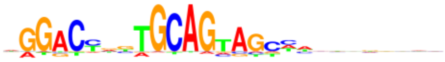
ZNF596	Q8TC21	KRAB	11	97.5	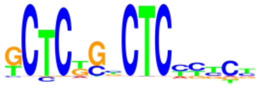
ZNF675*	Q8TD23	KRAB	12	105	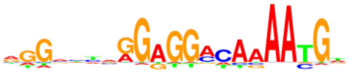
KLF14*	Q8TD94	none	3	936	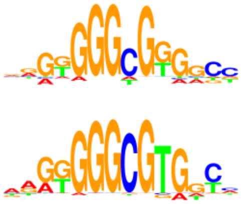 (PMID 23332764)
ZNF418	Q8TF45	KRAB	16	105	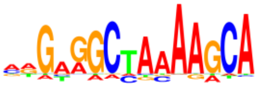
ZNF384	Q8TF68	none	8	733	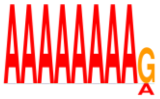

GTF3A	Q92664	none	9	320.5	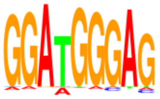
ZNF669*	Q96BR6	KRAB	9	6.6	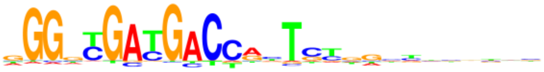
ZNF524	Q96C55	none	4	163.7	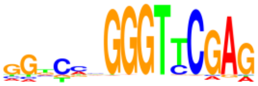 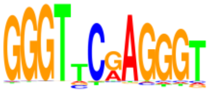 (PMID 23332764)
ZNF419	Q96HQ0	KRAB	11	105	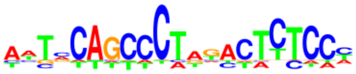
ZNF594	Q96JF6	none	21	105	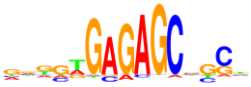
ZSCAN31*	Q96LW9	SCAN	6	179.2	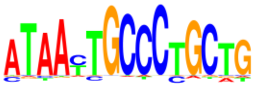
ZFP42	Q96MM3	none	4	936	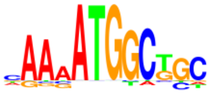
ZNF778	Q96MU6	KRAB	14	163.7	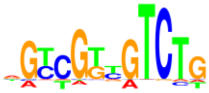
ZNF582	Q96NG8	KRAB	9	105	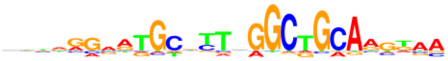
ZFP3	Q96NJ6	none	13	163.7	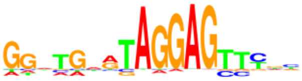
ZIM3	Q96PE6	KRAB	11	97.5	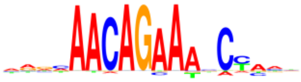
ZNF317*	Q96PQ6	KRAB	13	105	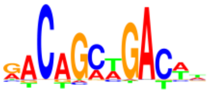
ZNF382*	Q96SR6	KRAB	9	105	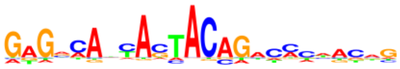

ZBTB18*	Q99592	BTB	4	429.6	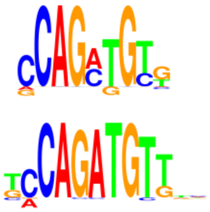 (PMID 16381825)
ZNF184	Q99676	KRAB	19	105	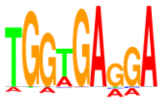
ZNF692*	Q9BU19	none	5	320.5	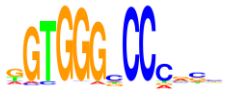
SCRT1	Q9BWW7	none	5	936	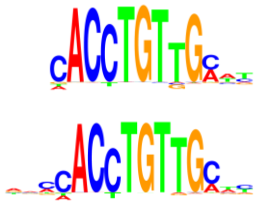 (PMID 23332764)
ZNF341	Q9BYN7	none	11	320.5	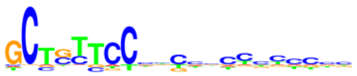
ZNF436	Q9C0F3	KRAB	12	163.7	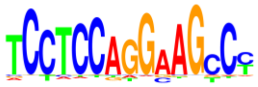
ZNF350	Q9GZX5	KRAB	8	105	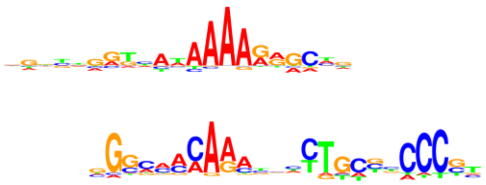 (PMID 23175603)
ZSCAN16	Q9H4T2	SCAN	4	163.7	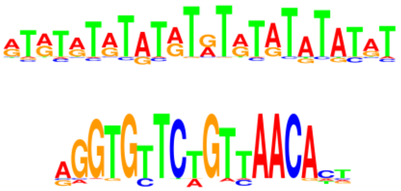 (PMID 23332764)
ZNF768	Q9H5H4	none	10	163.7	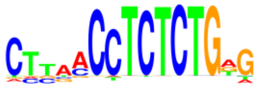

PATZ1	Q9HBE1	BTB	7	320.5	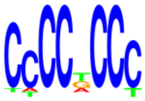 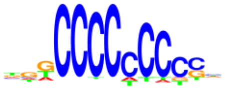 (PMID 16381825)
ZBTB26	Q9HCK0	BTB	4	355.7	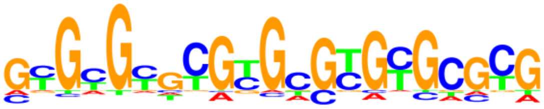
ZFP64	Q9NPA5	none	10	320.5	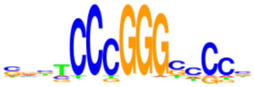
MYNN	Q9NPC7	BTB	8	320.5	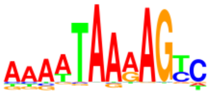
PRDM6	Q9NQX0	SET	3	936	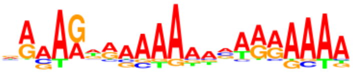
ZNF331	Q9NQX6	KRAB	12	105	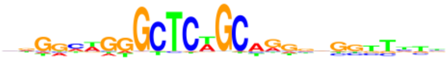
ZNF71*	Q9NQZ8	none	13	90.9	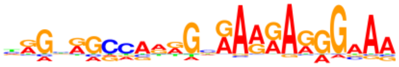
ZNF586	Q9NXT0	KRAB	8	105	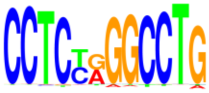
ZNF224	Q9NZL3	KRAB	18	163.7	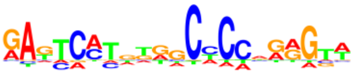
KLF15	Q9UIH9	none	3	936	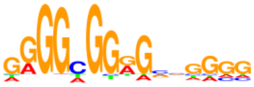 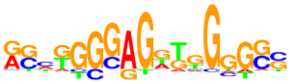 (PMID 23175603)
IKZF3	Q9UKT9	none	6	320.5	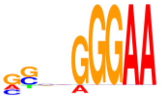
ZNF214	Q9UL59	KRAB	10	163.7	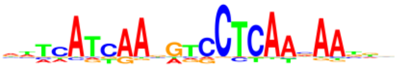

ZNF490	Q9ULM2	KRAB	13	43.1	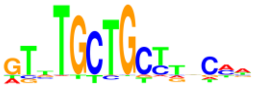
ZNF257	Q9Y2Q1	KRAB	11	97.5	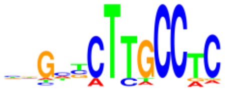
ZNF281	Q9Y2X9	none	4	320.5	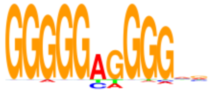
ZBTB12*	Q9Y330	BTB	4	429.6	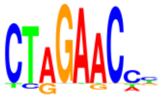
ZNF175	Q9Y473	KRAB	13	105	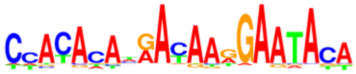
KLF12	Q9Y4X4	none	3	936	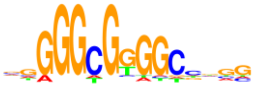 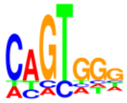 (PMID 16381825)
