# Supplementary material for: Multiparameter functional diversity of human C2H2 zinc finger proteins
Source: Genome Res. 2016 Dec;26(12):1742–52. doi: 10.1101/gr.209643.116 (PMC5131825; doi:10.1101/gr.209643.116)
Supplement: Supplemental Material [file supp_gr.209643.116_Supplemental_Figure_S7.pdf]

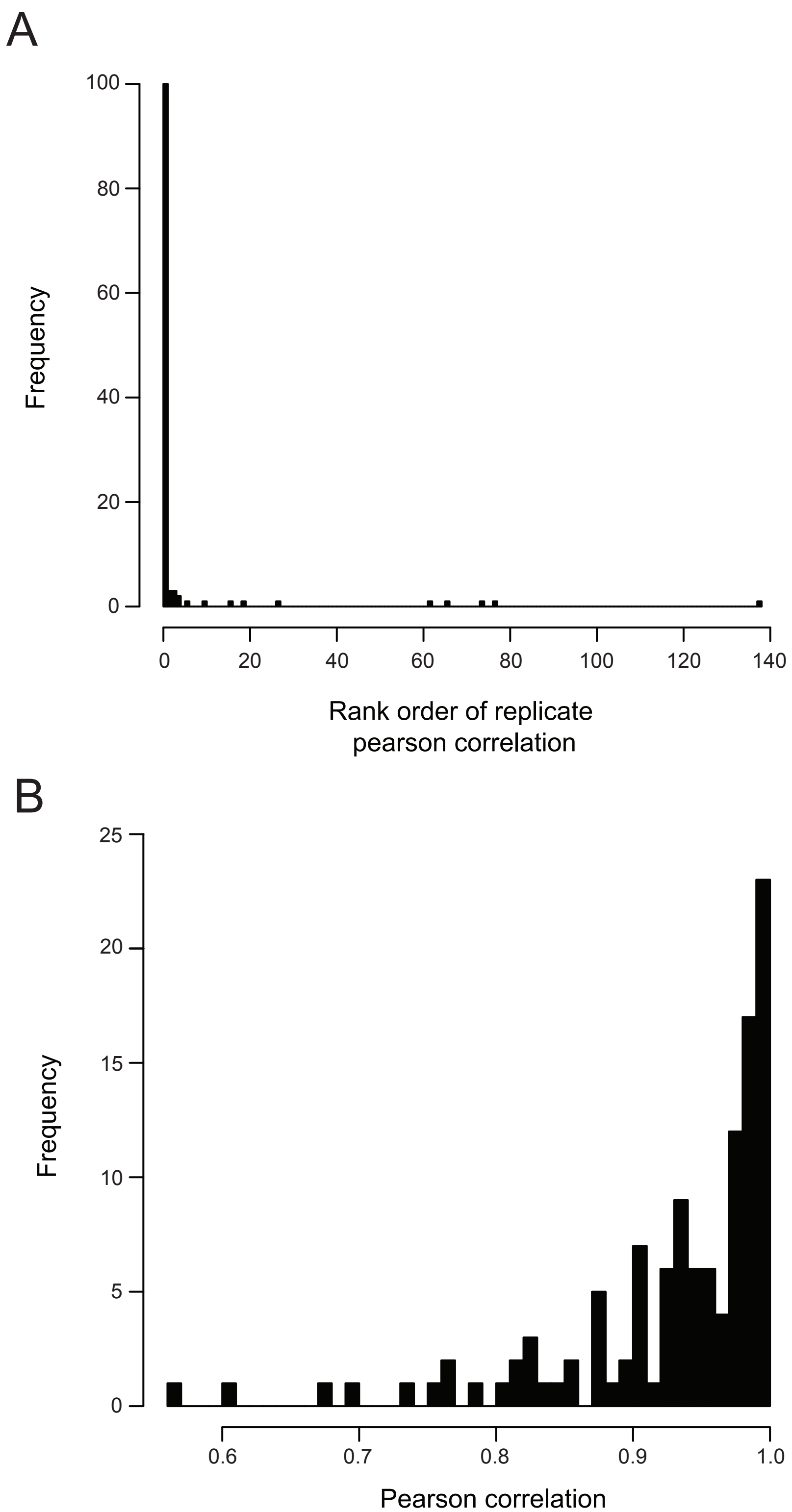

Supplemental Figure S7 (related to Figure 5): Reproducibility between AP-MS replicates.
(A) Distribution of rank orders of AP-MS replicate Pearson correlation compared to all other replicates based on the raw spectral counts from AP-MS results, using a matrix of 236 baits (each of the 118 in duplicate) x 344 prey proteins (the same set described in the text for PANTHER analysis). For example, a rank order of 1 for EGR2 indicates that EGR2 replicate 1 was more similar to EGR2 replicate 2 than all other 235 individual AP-MS experiments. (B) Distribution of Pearson correlations for each pair of AP-MS replicates for the same C2H2-ZFP, as in A. The correlation between replicates is above 90% for 93/118 C2H2-ZFs.
